# Measuring Pedestrian Collision Detection With Peripheral Field Loss and the Impact of Peripheral Prisms

**DOI:** 10.1167/tvst.7.5.1

**Published:** 2018-09-04

**Authors:** Cheng Qiu, Jae-Hyun Jung, Merve Tuccar-Burak, Lauren Spano, Robert Goldstein, Eli Peli

**Affiliations:** 1 Schepens Eye Research Institute, Massachusetts Eye and Ear, Department of Ophthalmology, Harvard Medical School, Boston, MA, USA

**Keywords:** peripheral field loss, tunnel vision, walking simulator, visual field expansion, retinitis pigmentosa, vision rehabilitation, prism

## Abstract

**Purpose:**

Peripheral field loss (PFL) due to retinitis pigmentosa, choroideremia, or glaucoma often results in a highly constricted residual central field, which makes it difficult for patients to avoid collision with approaching pedestrians. We developed a virtual environment to evaluate the ability of patients to detect pedestrians and judge potential collisions. We validated the system with both PFL patients and normally sighted subjects with simulated PFL. We also tested whether properly placed high-power prisms may improve pedestrian detection.

**Methods:**

A virtual park-like open space was rendered using a driving simulator (configured for walking speeds), and pedestrians in testing scenarios appeared within and outside the residual central field. Nine normally sighted subjects and eight PFL patients performed the pedestrian detection and collision judgment tasks. The performance of the subjects with simulated PFL was further evaluated with field of view expanding prisms.

**Results:**

The virtual system for testing pedestrian detection and collision judgment was validated. The performance of PFL patients and normally sighted subjects with simulated PFL were similar. The prisms for simulated PFL improved detection rates, reduced detection response times, and supported reasonable collision judgments in the prism-expanded field; detections and collision judgments in the residual central field were not influenced negatively by the prisms.

**Conclusions:**

The scenarios in a virtual environment are suitable for evaluating PFL and the impact of field of view expanding devices.

**Translational Relevance:**

This study validated an objective means to evaluate field expansion devices in reproducible near-real-life settings.

## Introduction

Peripheral field loss (PFL), sometimes called tunnel vision, is characterized by the severe concentric loss of peripheral vision, resulting in a limited residual central field.^1,2^ A residual central visual field of 20° or less in diameter is considered legal blindness in most jurisdictions.^3^ It can be caused by diseases such as retinitis pigmentosa (RP), choroideremia, or advanced glaucoma.^2^ The loss of peripheral vision often progresses gradually, becoming more severe over decades.^4,5^ Patients with PFL report difficulties with mobility, such as tripping over obstacles or uneven travel surfaces and bumping into people in crowded situations^6^; indeed, PFL has shown correlations with limited mobility performance.^7–11^ This paper addresses the difficulties faced by PFL patients in avoiding collisions with other pedestrians while walking in crowded open spaces such as transportation terminals, wide school corridors, and shopping malls, where the directions of pedestrian movement are not regulated as they are in office building corridors or along sidewalks.

With a normal visual field, visual cues about other pedestrians' movements can be utilized to estimate whether they pose any collision risk. Subsequent decisions on changing the trajectory or speed may then be made. We first analyze the visual information that may not be accessible to the patient. The direction of a pedestrian relative to the patient's heading is defined as the bearing angle,^1^ and this angle is equal to the visual eccentricity of the pedestrian in the perceived scene while the patient is gazing straight ahead, as is commonly the case during walking. The below two examples illustrate how the bearing (retinal eccentricity) and retinal image size (looming) of approaching pedestrians change in patients' visual field during the approach.

**Figure 1:**
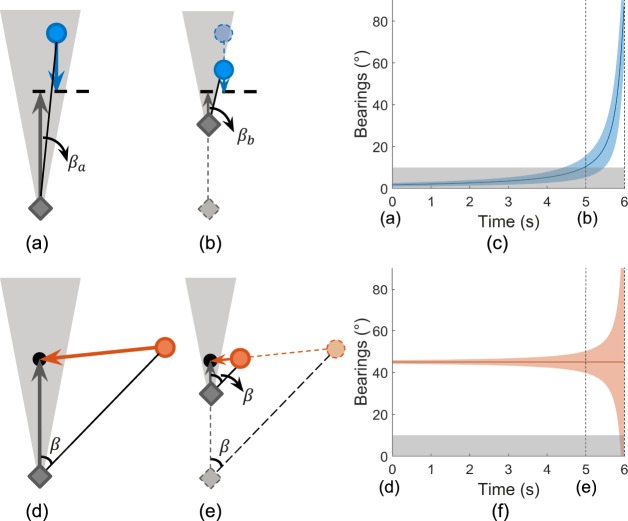
Near-collision (a–c) and center-to-center collision (d–f) that can be encountered by patients with PFL (illustrated as a gray diamond). (a) Top view of a near-collision when the two pedestrians are on sidewalks or in corridors, where their trajectories are parallel. The solid gray diamond and blue circle show the initial positions of the patient and another pedestrian, respectively. The angle formed by the gray and black solid lines, $\newcommand{\bialpha}{\boldsymbol{\alpha}}\newcommand{\bibeta}{\boldsymbol{\beta}}\newcommand{\bigamma}{\boldsymbol{\gamma}}\newcommand{\bidelta}{\boldsymbol{\delta}}\newcommand{\bivarepsilon}{\boldsymbol{\varepsilon}}\newcommand{\bizeta}{\boldsymbol{\zeta}}\newcommand{\bieta}{\boldsymbol{\eta}}\newcommand{\bitheta}{\boldsymbol{\theta}}\newcommand{\biiota}{\boldsymbol{\iota}}\newcommand{\bikappa}{\boldsymbol{\kappa}}\newcommand{\bilambda}{\boldsymbol{\lambda}}\newcommand{\bimu}{\boldsymbol{\mu}}\newcommand{\binu}{\boldsymbol{\nu}}\newcommand{\bixi}{\boldsymbol{\xi}}\newcommand{\biomicron}{\boldsymbol{\micron}}\newcommand{\bipi}{\boldsymbol{\pi}}\newcommand{\birho}{\boldsymbol{\rho}}\newcommand{\bisigma}{\boldsymbol{\sigma}}\newcommand{\bitau}{\boldsymbol{\tau}}\newcommand{\biupsilon}{\boldsymbol{\upsilon}}\newcommand{\biphi}{\boldsymbol{\phi}}\newcommand{\bichi}{\boldsymbol{\chi}}\newcommand{\bipsi}{\boldsymbol{\psi}}\newcommand{\biomega}{\boldsymbol{\omega}}{\beta _a}$, is the pedestrian's bearing angle relative to the patient's heading direction. The patient and pedestrian are presumed to reach where the arrows point after 6 seconds, where the distance between their central points becomes the closest (0.6 m). The shaded gray triangle illustrates the residual central field visible to a patient with a 20° diameter residual central field. (b) Top view of the patient and pedestrian pair 5 seconds after (a). The bearing angle changes to $\newcommand{\bialpha}{\boldsymbol{\alpha}}\newcommand{\bibeta}{\boldsymbol{\beta}}\newcommand{\bigamma}{\boldsymbol{\gamma}}\newcommand{\bidelta}{\boldsymbol{\delta}}\newcommand{\bivarepsilon}{\boldsymbol{\varepsilon}}\newcommand{\bizeta}{\boldsymbol{\zeta}}\newcommand{\bieta}{\boldsymbol{\eta}}\newcommand{\bitheta}{\boldsymbol{\theta}}\newcommand{\biiota}{\boldsymbol{\iota}}\newcommand{\bikappa}{\boldsymbol{\kappa}}\newcommand{\bilambda}{\boldsymbol{\lambda}}\newcommand{\bimu}{\boldsymbol{\mu}}\newcommand{\binu}{\boldsymbol{\nu}}\newcommand{\bixi}{\boldsymbol{\xi}}\newcommand{\biomicron}{\boldsymbol{\micron}}\newcommand{\bipi}{\boldsymbol{\pi}}\newcommand{\birho}{\boldsymbol{\rho}}\newcommand{\bisigma}{\boldsymbol{\sigma}}\newcommand{\bitau}{\boldsymbol{\tau}}\newcommand{\biupsilon}{\boldsymbol{\upsilon}}\newcommand{\biphi}{\boldsymbol{\phi}}\newcommand{\bichi}{\boldsymbol{\chi}}\newcommand{\bipsi}{\boldsymbol{\psi}}\newcommand{\biomega}{\boldsymbol{\omega}}{\beta _b}$, which gradually moves out of the patient's residual central field (the shaded gray triangle). (c) The changes of the pedestrian's bearing span relative to the patient's heading direction as a function of time ((a) to (b) and to the 6th second). The gray area indicates the patient's visible field on the right side over time. (d) A center-to-center collision case, which is likely in an open space where pedestrians' paths are less regulated. The pedestrian shown as an orange circle is outside the patient's residual central field. The black dot indicates the collision point where the patient (gray diamond) and pedestrian (orange circle) would arrive simultaneously. (e) Top view of the same patient and pedestrian pair 5 seconds after (d). (f) The bearing span as a function of time relative to the field of the patient shown in gray. The pedestrian is only visible to the patient's residual central field after 5.8 seconds.

On sidewalks or in corridors, the oncoming pedestrians' walking trajectories tend to be parallel to the patients. Figures 1a and 1b show an example with two pedestrians approaching each other on parallel paths. The example assumes that the pedestrian (shown as a gray diamond) is a patient with 20° diameter residual central field. The approaching pedestrian is shown as a blue circle. Assuming an average shoulder width of about 0.6 m, the closest distance between the central points of two pedestrians needs to be more than 0.6 m to avoid a collision. In this example, the closest distance between the patient's and pedestrian's central points is 0.6 m. This may result in a socially unacceptable close proximity, which we call brushing or *near-collision*. The change in bearing angle (*β*) as a function of time is shown in Figure 1c, assuming the patient's gaze is maintained straight ahead: the solid blue line indicates the bearing dynamics of the pedestrian's central point, and the shaded blue area represents the bearing span over time of an assumed cylinder-shaped pedestrian with a 0.6-m diameter. Figure 1c shows that the blue pedestrian looms and stays within the residual central field of the patient (shaded in gray) for about 5 seconds. This (near) colliding pedestrian would appear within the patient's residual central field and thus be easily detected and attended to early on to avoid the collision.

In the second example, an open space scenario (Figs. 1d, 1e), the pedestrian approaches the patient from the side, and their geometrical centers are on a collision course contacting at the black dot (*center-to-center collision*). The orange circular pedestrian in Figure 1d approaches the patient (depicted as a gray diamond) at a bearing angle (*β*) of 45°, and they both maintain a constant (though not necessarily equal) speed and direction. Because they are on a collision course, the triangles formed by the patient, the orange pedestrian, and the collision point are similar triangles (Fig. 1e), which means that the bearing angle of the pedestrian relative to the patient's heading direction stays constant (the orange horizontal solid line in Fig. 1f). The shaded orange area shows the bearing span over time when the body width is considered (Fig. 1f). Different from the blue pedestrian (Fig. 1c), the orange span stays outside the patient's residual central field most of the time until right before the collision happens (assuming forward gaze). This is consistent with the anecdotal account often given by patients that pedestrians seem to *pop up*, leaving the patient with no time to respond. Such cases are most likely to happen in crowded open spaces where pedestrians' trajectories are highly variable and less regulated.

Patients with PFL are often instructed in orientation and mobility training to compensate for their impairment through scanning. However, scanning per se was found to be ineffective especially when walking.^12^ Forward-gazing may be crucial for monitoring locomotion, especially given a limited visual field,^13–15^ and spending a lot of time on scanning has been suggested to be unsafe for patients.^16^ Patients with PFL in fact show limited scanning, of about the same magnitude as normally sighted pedestrians, while walking indoors or outdoors.^17,18^ To summarize, for patients with PFL, potential collisions with pedestrians on sidewalks or corridors may be easy to detect with their intact central vision, but those coming from the side (e.g., in open spaces) are likely to be missed.

Here we report on the development and validation of virtual reality (VR) walking scenarios as a tool to objectively evaluate PFL patients' ability to avoid collisions with other pedestrians with and without visual aids or other treatment (e.g., scanning training). The virtual scenarios were rendered using a driving simulator configured for walking speeds, in which the participants walk virtually in a park. They were asked to perform two consecutive tasks for each appearing pedestrian—pedestrian detection and collision judgment—while controlling their path through the park. The virtual system was verified in nine normally sighted subjects with simulated PFL (binocular 20° diameter residual central fields using masked head-mounted goggles) and eight PFL patients.

We are also developing novel prism glasses^2,19^ as visual aids for PFL patients and testing their effect on patients' performance using this virtual system. The prism glasses aim to expand patients' field of view (the portion of the scene that falls on patients' functioning retina) using prisms to shift the previously unseen portion of the scene onto the functioning retina.^19^ Peripheral prism glasses have been used to help patients detect objects in their blind fields through shifted views.^20^ Peripheral Peli prisms, developed for homonymous hemianopia (HH), are placed in the upper and lower periphery to provide field of view expansion without causing central binocular double vision. One component of double vision, the appearance of two different objects at the same perceived direction, is binocular visual confusion, which is necessary for field expansion but not acceptable for central vision.^21,22^ Peripheral prisms were therefore found to be beneficial in obstacle avoidance when walking.^23^

To decide which portion of the scene to shift using the prisms, we asked where in the visual field potentially colliding pedestrians in crowded open spaces (e.g., Fig. 1d) are more likely to occur. Peli et al.^1^ addressed this question by calculating the risk density of collisions at different bearings of pedestrians relative to the patient's heading. They found that, given realistic assumptions on speed ranges and assuming pedestrians at all locations head in all directions with equal probability, the collision risk density peaked at a bearing angle of about 45°. Based on their model, pedestrians approaching at low-bearing angles (more centrally) posed a lower risk for collision; for example, −10° to 10° bearings (negative sign for the left side) account for only 3% of the total risk in such open space. They proposed creating artificial visual islands in the periphery using prisms (prism-expanded fields). For example, artificial visual islands 20° diameter wide centered at 30° eccentricity (theoretically achievable using 57 prism diopter [Δ] [≈30°] prisms) on both sides would enable about 31% of the total risk to be monitored.^1^ It is also important to assure that while expanding the peripheral field of view, disturbance to patients' residual central visual field is avoided or minimized.

Furthermore, oblique peripheral prisms were applied, shifting the images both horizontally and vertically,^24^ moving the laterally expanded fields of view up or down to about eye level. Without the vertical shift, the user may not detect an approaching pedestrian through the upper prism unless the pedestrian is much taller; the lower prism also may be less effective for pedestrian detection due to showing mostly lower parts of the pedestrian or ground depending on the distance.

The effect of the prisms is often measured using standard perimetry, and their real-life impact on mobility is usually evaluated subjectively through survey or questionnaires. However, standard perimetry can only indicate the field of view given a fixed gaze position under optimal viewing conditions (e.g., participants detect one simple target on a blank background as the sole task). Therefore, such measurements are inadequate to predict the prisms' impact in complex real-life mobility situations. An objective evaluation of performance is necessary, especially when high-power prisms are prescribed, since various side effects, such as prism distortions, total internal reflection (TIR), and possible spurious reflections,^25^ may limit the effectiveness of the prisms. The possible impact of these limitations may go unnoticed by the patients, as pedestrian collision is unpredictable in real life and the frequency is low (but may be critical when it happens). More importantly, the patients will never know about missed detections if other pedestrians corrected their paths or stopped to avoid the collision. Therefore, it is impractical to evaluate with questionnaires or to test in real life. The effects of the peripheral prisms on pedestrian detection by patients with HH while driving were evaluated in a driving simulator^26^ and in an on-road study,^27^ which better reflect their real-life impact.

Using the novel VR test environment, we conducted a first evaluation of the field of view expanding prisms for PFL. We used a prism design modified from the Peli peripheral prisms for HH^21^ to address the PFL situation.^19^ The impact of the prisms within both the prism-expanded fields and the residual central fields were examined with simulated PFL in normally sighted subjects.

## Methods

This study was designed to create and validate a VR test environment, in which the impact of PFL on pedestrian detection and collision judgment performance can be objectively evaluated. Such evaluation would be particularly useful in comparing performance with various treatment options (devices and training). Here we included a first evaluation of the impact of peripheral prisms with simulated PFL as a way of demonstrating our test systems. Part I describes the virtual walking scenarios, which included multiple pedestrians appearing at various visual eccentricities and approaching the participant's path on both collision and noncollision courses. Part II first outlines a prospective design for peripheral prism placement on glasses for PFL, and then details the adaptation to the goggles simulating PFL. Lastly, Part III presents the experimental procedure and data analyses.

### Part I. Simulator Scenarios for Evaluating Pedestrian Detection and Collision Judgment

#### Virtual Environment

**Figure 2:**
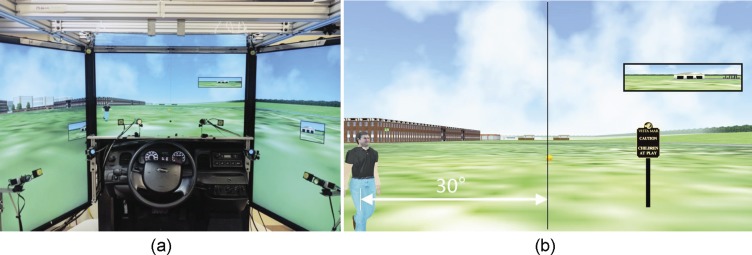
Simulated walking in an open space environment. (a) An open park scene rendered on the driving simulator (only three out of five screens are shown). (b) A front screen image showing a pedestrian approaching from the left at 30° bearing. The string marking the center of the screen for aligning the heading direction is illustrated using a black vertical line. The participant maintains the planned walking path by steering to align the intended path traced by the basketball (small orange circle) with the string.

An open space walking environment was simulated using a driving simulator model LE1500 (FAAC, Inc., Ann Arbor, MI), which provides a 225° field of view with five screens (Fig. 2a) for participants seated 735 mm from the front screen. The scenarios were scripted using the software Scenario Toolbox, supplied by FAAC. Participants “walked” in an open park environment with green grass grounds without other obstacles. Roads, buildings, and trees were visible in the background. The simulated vehicle was a bicycle, which provided a wider open view as would be available for a pedestrian rather than the limited window view that is blocked by the car interior seen in driving simulations. The bicycles' speed was set at a fixed rapid walking speed, 1.94 m/s. Gas and brake pedals were disabled. Seated participants controlled only their “walking” direction using the steering wheel. Active control was needed to engage the participants' attention and keep them gazing forward, as is the case during walking. An orange basketball rolling ahead of participants was used to mark the path, guiding their walking directions, and they were instructed to keep the ball aligned with a string marking the center of the front screen (Fig. 2b). Without such a reference, participants with PFL (real or simulated) may not notice if they drift from the screen center^28^ and the assumed path that the pedestrian eccentricities depended upon.

Each scenario lasted about 8 minutes and included 20 segments with slightly varying walking directions. Each segment was about 45 m (23 seconds) long. The orange basketball path turned by a small angle at the beginning of each segment, and participants were allowed 15 ± 2 m (about 8 seconds) to realign with the basketball before a programmed pedestrian appeared (Fig. 2b). Only gradual turns (≤5°) were applied to ensure easy alignment so as to minimize the differences between programmed and actual pedestrian bearings. The participants were told to maintain the basketball and string alignment, but they were not required to stare at it all the time. Only one pedestrian appeared in each segment, during which the participant and pedestrian were both programmed to maintain constant (though can be different) speeds and fixed directions. Across the segments, the pedestrian's speed varied according to the designed path, and pedestrians approaching at a fast speed were rendered to appear running. The participants were instructed to press the horn once to indicate pedestrian detection, and then again for their judgment of collision. Pedestrians were scripted to disappear 0.1 second before they reached the participant's path, to avoid actual collisions or bypasses, but participants were instructed to make their decisions about collisions based on the assumption that pedestrians would continue moving on the same path. No feedback was given after the responses.

#### Design of Pedestrian Paths

**Figure 3:**
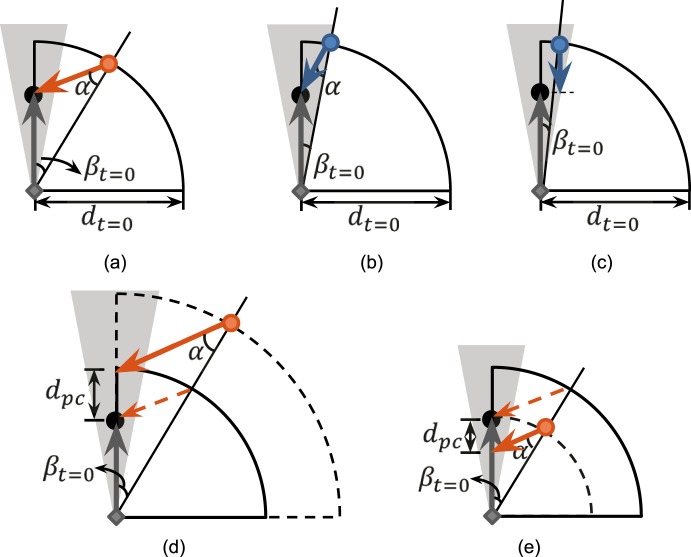
Diagrams of the various trials with pedestrians appearing initially at various bearings ($\newcommand{\bialpha}{\boldsymbol{\alpha}}\newcommand{\bibeta}{\boldsymbol{\beta}}\newcommand{\bigamma}{\boldsymbol{\gamma}}\newcommand{\bidelta}{\boldsymbol{\delta}}\newcommand{\bivarepsilon}{\boldsymbol{\varepsilon}}\newcommand{\bizeta}{\boldsymbol{\zeta}}\newcommand{\bieta}{\boldsymbol{\eta}}\newcommand{\bitheta}{\boldsymbol{\theta}}\newcommand{\biiota}{\boldsymbol{\iota}}\newcommand{\bikappa}{\boldsymbol{\kappa}}\newcommand{\bilambda}{\boldsymbol{\lambda}}\newcommand{\bimu}{\boldsymbol{\mu}}\newcommand{\binu}{\boldsymbol{\nu}}\newcommand{\bixi}{\boldsymbol{\xi}}\newcommand{\biomicron}{\boldsymbol{\micron}}\newcommand{\bipi}{\boldsymbol{\pi}}\newcommand{\birho}{\boldsymbol{\rho}}\newcommand{\bisigma}{\boldsymbol{\sigma}}\newcommand{\bitau}{\boldsymbol{\tau}}\newcommand{\biupsilon}{\boldsymbol{\upsilon}}\newcommand{\biphi}{\boldsymbol{\phi}}\newcommand{\bichi}{\boldsymbol{\chi}}\newcommand{\bipsi}{\boldsymbol{\psi}}\newcommand{\biomega}{\boldsymbol{\omega}}{\beta _{t = 0}}$) and crossing the participant's path at various distances from the participant ($\newcommand{\bialpha}{\boldsymbol{\alpha}}\newcommand{\bibeta}{\boldsymbol{\beta}}\newcommand{\bigamma}{\boldsymbol{\gamma}}\newcommand{\bidelta}{\boldsymbol{\delta}}\newcommand{\bivarepsilon}{\boldsymbol{\varepsilon}}\newcommand{\bizeta}{\boldsymbol{\zeta}}\newcommand{\bieta}{\boldsymbol{\eta}}\newcommand{\bitheta}{\boldsymbol{\theta}}\newcommand{\biiota}{\boldsymbol{\iota}}\newcommand{\bikappa}{\boldsymbol{\kappa}}\newcommand{\bilambda}{\boldsymbol{\lambda}}\newcommand{\bimu}{\boldsymbol{\mu}}\newcommand{\binu}{\boldsymbol{\nu}}\newcommand{\bixi}{\boldsymbol{\xi}}\newcommand{\biomicron}{\boldsymbol{\micron}}\newcommand{\bipi}{\boldsymbol{\pi}}\newcommand{\birho}{\boldsymbol{\rho}}\newcommand{\bisigma}{\boldsymbol{\sigma}}\newcommand{\bitau}{\boldsymbol{\tau}}\newcommand{\biupsilon}{\boldsymbol{\upsilon}}\newcommand{\biphi}{\boldsymbol{\phi}}\newcommand{\bichi}{\boldsymbol{\chi}}\newcommand{\bipsi}{\boldsymbol{\psi}}\newcommand{\biomega}{\boldsymbol{\omega}}{d_{pc}}$). The participant is shown as a gray diamond and the pedestrian is a blue circle if its bearing is within the simulated residual central field or orange circle if it is outside. The shaded triangle in light gray indicates the simulated residual central field with 20° diameter. (a) A center-to-center collision with $\newcommand{\bialpha}{\boldsymbol{\alpha}}\newcommand{\bibeta}{\boldsymbol{\beta}}\newcommand{\bigamma}{\boldsymbol{\gamma}}\newcommand{\bidelta}{\boldsymbol{\delta}}\newcommand{\bivarepsilon}{\boldsymbol{\varepsilon}}\newcommand{\bizeta}{\boldsymbol{\zeta}}\newcommand{\bieta}{\boldsymbol{\eta}}\newcommand{\bitheta}{\boldsymbol{\theta}}\newcommand{\biiota}{\boldsymbol{\iota}}\newcommand{\bikappa}{\boldsymbol{\kappa}}\newcommand{\bilambda}{\boldsymbol{\lambda}}\newcommand{\bimu}{\boldsymbol{\mu}}\newcommand{\binu}{\boldsymbol{\nu}}\newcommand{\bixi}{\boldsymbol{\xi}}\newcommand{\biomicron}{\boldsymbol{\micron}}\newcommand{\bipi}{\boldsymbol{\pi}}\newcommand{\birho}{\boldsymbol{\rho}}\newcommand{\bisigma}{\boldsymbol{\sigma}}\newcommand{\bitau}{\boldsymbol{\tau}}\newcommand{\biupsilon}{\boldsymbol{\upsilon}}\newcommand{\biphi}{\boldsymbol{\phi}}\newcommand{\bichi}{\boldsymbol{\chi}}\newcommand{\bipsi}{\boldsymbol{\psi}}\newcommand{\biomega}{\boldsymbol{\omega}}{\beta _{t = 0}} = 30^{\circ}$. The participant and the pedestrian arrive at the collision point simultaneously ($\newcommand{\bialpha}{\boldsymbol{\alpha}}\newcommand{\bibeta}{\boldsymbol{\beta}}\newcommand{\bigamma}{\boldsymbol{\gamma}}\newcommand{\bidelta}{\boldsymbol{\delta}}\newcommand{\bivarepsilon}{\boldsymbol{\varepsilon}}\newcommand{\bizeta}{\boldsymbol{\zeta}}\newcommand{\bieta}{\boldsymbol{\eta}}\newcommand{\bitheta}{\boldsymbol{\theta}}\newcommand{\biiota}{\boldsymbol{\iota}}\newcommand{\bikappa}{\boldsymbol{\kappa}}\newcommand{\bilambda}{\boldsymbol{\lambda}}\newcommand{\bimu}{\boldsymbol{\mu}}\newcommand{\binu}{\boldsymbol{\nu}}\newcommand{\bixi}{\boldsymbol{\xi}}\newcommand{\biomicron}{\boldsymbol{\micron}}\newcommand{\bipi}{\boldsymbol{\pi}}\newcommand{\birho}{\boldsymbol{\rho}}\newcommand{\bisigma}{\boldsymbol{\sigma}}\newcommand{\bitau}{\boldsymbol{\tau}}\newcommand{\biupsilon}{\boldsymbol{\upsilon}}\newcommand{\biphi}{\boldsymbol{\phi}}\newcommand{\bichi}{\boldsymbol{\chi}}\newcommand{\bipsi}{\boldsymbol{\psi}}\newcommand{\biomega}{\boldsymbol{\omega}}{d_{pc}} = 0$). The pedestrian is invisible to the participant's residual central field most of the time. (b) A center-to-center collision with $\newcommand{\bialpha}{\boldsymbol{\alpha}}\newcommand{\bibeta}{\boldsymbol{\beta}}\newcommand{\bigamma}{\boldsymbol{\gamma}}\newcommand{\bidelta}{\boldsymbol{\delta}}\newcommand{\bivarepsilon}{\boldsymbol{\varepsilon}}\newcommand{\bizeta}{\boldsymbol{\zeta}}\newcommand{\bieta}{\boldsymbol{\eta}}\newcommand{\bitheta}{\boldsymbol{\theta}}\newcommand{\biiota}{\boldsymbol{\iota}}\newcommand{\bikappa}{\boldsymbol{\kappa}}\newcommand{\bilambda}{\boldsymbol{\lambda}}\newcommand{\bimu}{\boldsymbol{\mu}}\newcommand{\binu}{\boldsymbol{\nu}}\newcommand{\bixi}{\boldsymbol{\xi}}\newcommand{\biomicron}{\boldsymbol{\micron}}\newcommand{\bipi}{\boldsymbol{\pi}}\newcommand{\birho}{\boldsymbol{\rho}}\newcommand{\bisigma}{\boldsymbol{\sigma}}\newcommand{\bitau}{\boldsymbol{\tau}}\newcommand{\biupsilon}{\boldsymbol{\upsilon}}\newcommand{\biphi}{\boldsymbol{\phi}}\newcommand{\bichi}{\boldsymbol{\chi}}\newcommand{\bipsi}{\boldsymbol{\psi}}\newcommand{\biomega}{\boldsymbol{\omega}}{\beta _{t = 0}} = 10^{\circ}$. (c) The participant and the pedestrian walk on parallel paths but toward each other with $\newcommand{\bialpha}{\boldsymbol{\alpha}}\newcommand{\bibeta}{\boldsymbol{\beta}}\newcommand{\bigamma}{\boldsymbol{\gamma}}\newcommand{\bidelta}{\boldsymbol{\delta}}\newcommand{\bivarepsilon}{\boldsymbol{\varepsilon}}\newcommand{\bizeta}{\boldsymbol{\zeta}}\newcommand{\bieta}{\boldsymbol{\eta}}\newcommand{\bitheta}{\boldsymbol{\theta}}\newcommand{\biiota}{\boldsymbol{\iota}}\newcommand{\bikappa}{\boldsymbol{\kappa}}\newcommand{\bilambda}{\boldsymbol{\lambda}}\newcommand{\bimu}{\boldsymbol{\mu}}\newcommand{\binu}{\boldsymbol{\nu}}\newcommand{\bixi}{\boldsymbol{\xi}}\newcommand{\biomicron}{\boldsymbol{\micron}}\newcommand{\bipi}{\boldsymbol{\pi}}\newcommand{\birho}{\boldsymbol{\rho}}\newcommand{\bisigma}{\boldsymbol{\sigma}}\newcommand{\bitau}{\boldsymbol{\tau}}\newcommand{\biupsilon}{\boldsymbol{\upsilon}}\newcommand{\biphi}{\boldsymbol{\phi}}\newcommand{\bichi}{\boldsymbol{\chi}}\newcommand{\bipsi}{\boldsymbol{\psi}}\newcommand{\biomega}{\boldsymbol{\omega}}{\beta _{t = 0}} = 5^{\circ}$ and the closest distance 1.69 m (noncollision). In (b) and (c), the pedestrian is initially visible within the participant's residual central field. (d) A near- or noncollision trial with $\newcommand{\bialpha}{\boldsymbol{\alpha}}\newcommand{\bibeta}{\boldsymbol{\beta}}\newcommand{\bigamma}{\boldsymbol{\gamma}}\newcommand{\bidelta}{\boldsymbol{\delta}}\newcommand{\bivarepsilon}{\boldsymbol{\varepsilon}}\newcommand{\bizeta}{\boldsymbol{\zeta}}\newcommand{\bieta}{\boldsymbol{\eta}}\newcommand{\bitheta}{\boldsymbol{\theta}}\newcommand{\biiota}{\boldsymbol{\iota}}\newcommand{\bikappa}{\boldsymbol{\kappa}}\newcommand{\bilambda}{\boldsymbol{\lambda}}\newcommand{\bimu}{\boldsymbol{\mu}}\newcommand{\binu}{\boldsymbol{\nu}}\newcommand{\bixi}{\boldsymbol{\xi}}\newcommand{\biomicron}{\boldsymbol{\micron}}\newcommand{\bipi}{\boldsymbol{\pi}}\newcommand{\birho}{\boldsymbol{\rho}}\newcommand{\bisigma}{\boldsymbol{\sigma}}\newcommand{\bitau}{\boldsymbol{\tau}}\newcommand{\biupsilon}{\boldsymbol{\upsilon}}\newcommand{\biphi}{\boldsymbol{\phi}}\newcommand{\bichi}{\boldsymbol{\chi}}\newcommand{\bipsi}{\boldsymbol{\psi}}\newcommand{\biomega}{\boldsymbol{\omega}}{d_{pc}} \gt 0$, where the pedestrian passes in front of the participant. The corresponding condition with the center-to-center collision is shown with the dashed orange line as a comparison. Given the same initial bearing ($\newcommand{\bialpha}{\boldsymbol{\alpha}}\newcommand{\bibeta}{\boldsymbol{\beta}}\newcommand{\bigamma}{\boldsymbol{\gamma}}\newcommand{\bidelta}{\boldsymbol{\delta}}\newcommand{\bivarepsilon}{\boldsymbol{\varepsilon}}\newcommand{\bizeta}{\boldsymbol{\zeta}}\newcommand{\bieta}{\boldsymbol{\eta}}\newcommand{\bitheta}{\boldsymbol{\theta}}\newcommand{\biiota}{\boldsymbol{\iota}}\newcommand{\bikappa}{\boldsymbol{\kappa}}\newcommand{\bilambda}{\boldsymbol{\lambda}}\newcommand{\bimu}{\boldsymbol{\mu}}\newcommand{\binu}{\boldsymbol{\nu}}\newcommand{\bixi}{\boldsymbol{\xi}}\newcommand{\biomicron}{\boldsymbol{\micron}}\newcommand{\bipi}{\boldsymbol{\pi}}\newcommand{\birho}{\boldsymbol{\rho}}\newcommand{\bisigma}{\boldsymbol{\sigma}}\newcommand{\bitau}{\boldsymbol{\tau}}\newcommand{\biupsilon}{\boldsymbol{\upsilon}}\newcommand{\biphi}{\boldsymbol{\phi}}\newcommand{\bichi}{\boldsymbol{\chi}}\newcommand{\bipsi}{\boldsymbol{\psi}}\newcommand{\biomega}{\boldsymbol{\omega}}{\beta _{t = 0}}$), the same pedestrian heading ($\newcommand{\bialpha}{\boldsymbol{\alpha}}\newcommand{\bibeta}{\boldsymbol{\beta}}\newcommand{\bigamma}{\boldsymbol{\gamma}}\newcommand{\bidelta}{\boldsymbol{\delta}}\newcommand{\bivarepsilon}{\boldsymbol{\varepsilon}}\newcommand{\bizeta}{\boldsymbol{\zeta}}\newcommand{\bieta}{\boldsymbol{\eta}}\newcommand{\bitheta}{\boldsymbol{\theta}}\newcommand{\biiota}{\boldsymbol{\iota}}\newcommand{\bikappa}{\boldsymbol{\kappa}}\newcommand{\bilambda}{\boldsymbol{\lambda}}\newcommand{\bimu}{\boldsymbol{\mu}}\newcommand{\binu}{\boldsymbol{\nu}}\newcommand{\bixi}{\boldsymbol{\xi}}\newcommand{\biomicron}{\boldsymbol{\micron}}\newcommand{\bipi}{\boldsymbol{\pi}}\newcommand{\birho}{\boldsymbol{\rho}}\newcommand{\bisigma}{\boldsymbol{\sigma}}\newcommand{\bitau}{\boldsymbol{\tau}}\newcommand{\biupsilon}{\boldsymbol{\upsilon}}\newcommand{\biphi}{\boldsymbol{\phi}}\newcommand{\bichi}{\boldsymbol{\chi}}\newcommand{\bipsi}{\boldsymbol{\psi}}\newcommand{\biomega}{\boldsymbol{\omega}}\alpha$) is used for various path crossing distances. (e) $\newcommand{\bialpha}{\boldsymbol{\alpha}}\newcommand{\bibeta}{\boldsymbol{\beta}}\newcommand{\bigamma}{\boldsymbol{\gamma}}\newcommand{\bidelta}{\boldsymbol{\delta}}\newcommand{\bivarepsilon}{\boldsymbol{\varepsilon}}\newcommand{\bizeta}{\boldsymbol{\zeta}}\newcommand{\bieta}{\boldsymbol{\eta}}\newcommand{\bitheta}{\boldsymbol{\theta}}\newcommand{\biiota}{\boldsymbol{\iota}}\newcommand{\bikappa}{\boldsymbol{\kappa}}\newcommand{\bilambda}{\boldsymbol{\lambda}}\newcommand{\bimu}{\boldsymbol{\mu}}\newcommand{\binu}{\boldsymbol{\nu}}\newcommand{\bixi}{\boldsymbol{\xi}}\newcommand{\biomicron}{\boldsymbol{\micron}}\newcommand{\bipi}{\boldsymbol{\pi}}\newcommand{\birho}{\boldsymbol{\rho}}\newcommand{\bisigma}{\boldsymbol{\sigma}}\newcommand{\bitau}{\boldsymbol{\tau}}\newcommand{\biupsilon}{\boldsymbol{\upsilon}}\newcommand{\biphi}{\boldsymbol{\phi}}\newcommand{\bichi}{\boldsymbol{\chi}}\newcommand{\bipsi}{\boldsymbol{\psi}}\newcommand{\biomega}{\boldsymbol{\omega}}{d_{pc}} \lt 0$, where the pedestrian passes behind the participant.

For each segment/trial, the participant's path was guided by the basketball. To represent the variety of pedestrian dynamics that patients face in real life, a few approaching pedestrian trajectories were included. This variability was also used to evaluate participants' sensitivity to the different pedestrians' dynamics; that is, the participants would be expected to report collisions more often for those pedestrians actually getting closer to them. Such responses would further add to the face validity of the testing system. In most trials, the pedestrian's path crossed the participant's path. We defined the distance between the participant and the pedestrian when their paths crossed as $\newcommand{\bialpha}{\boldsymbol{\alpha}}\newcommand{\bibeta}{\boldsymbol{\beta}}\newcommand{\bigamma}{\boldsymbol{\gamma}}\newcommand{\bidelta}{\boldsymbol{\delta}}\newcommand{\bivarepsilon}{\boldsymbol{\varepsilon}}\newcommand{\bizeta}{\boldsymbol{\zeta}}\newcommand{\bieta}{\boldsymbol{\eta}}\newcommand{\bitheta}{\boldsymbol{\theta}}\newcommand{\biiota}{\boldsymbol{\iota}}\newcommand{\bikappa}{\boldsymbol{\kappa}}\newcommand{\bilambda}{\boldsymbol{\lambda}}\newcommand{\bimu}{\boldsymbol{\mu}}\newcommand{\binu}{\boldsymbol{\nu}}\newcommand{\bixi}{\boldsymbol{\xi}}\newcommand{\biomicron}{\boldsymbol{\micron}}\newcommand{\bipi}{\boldsymbol{\pi}}\newcommand{\birho}{\boldsymbol{\rho}}\newcommand{\bisigma}{\boldsymbol{\sigma}}\newcommand{\bitau}{\boldsymbol{\tau}}\newcommand{\biupsilon}{\boldsymbol{\upsilon}}\newcommand{\biphi}{\boldsymbol{\phi}}\newcommand{\bichi}{\boldsymbol{\chi}}\newcommand{\bipsi}{\boldsymbol{\psi}}\newcommand{\biomega}{\boldsymbol{\omega}}{d_{pc}}$. A center-to-center collision would occur if and when $\newcommand{\bialpha}{\boldsymbol{\alpha}}\newcommand{\bibeta}{\boldsymbol{\beta}}\newcommand{\bigamma}{\boldsymbol{\gamma}}\newcommand{\bidelta}{\boldsymbol{\delta}}\newcommand{\bivarepsilon}{\boldsymbol{\varepsilon}}\newcommand{\bizeta}{\boldsymbol{\zeta}}\newcommand{\bieta}{\boldsymbol{\eta}}\newcommand{\bitheta}{\boldsymbol{\theta}}\newcommand{\biiota}{\boldsymbol{\iota}}\newcommand{\bikappa}{\boldsymbol{\kappa}}\newcommand{\bilambda}{\boldsymbol{\lambda}}\newcommand{\bimu}{\boldsymbol{\mu}}\newcommand{\binu}{\boldsymbol{\nu}}\newcommand{\bixi}{\boldsymbol{\xi}}\newcommand{\biomicron}{\boldsymbol{\micron}}\newcommand{\bipi}{\boldsymbol{\pi}}\newcommand{\birho}{\boldsymbol{\rho}}\newcommand{\bisigma}{\boldsymbol{\sigma}}\newcommand{\bitau}{\boldsymbol{\tau}}\newcommand{\biupsilon}{\boldsymbol{\upsilon}}\newcommand{\biphi}{\boldsymbol{\phi}}\newcommand{\bichi}{\boldsymbol{\chi}}\newcommand{\bipsi}{\boldsymbol{\psi}}\newcommand{\biomega}{\boldsymbol{\omega}}{d_{pc}} = 0$ (Figs. 3a, 3b). To allow sufficient time for responding to a potential collision, the time when their paths may cross was set to 6 seconds after the pedestrian appeared. The position of the participant at $\newcommand{\bialpha}{\boldsymbol{\alpha}}\newcommand{\bibeta}{\boldsymbol{\beta}}\newcommand{\bigamma}{\boldsymbol{\gamma}}\newcommand{\bidelta}{\boldsymbol{\delta}}\newcommand{\bivarepsilon}{\boldsymbol{\varepsilon}}\newcommand{\bizeta}{\boldsymbol{\zeta}}\newcommand{\bieta}{\boldsymbol{\eta}}\newcommand{\bitheta}{\boldsymbol{\theta}}\newcommand{\biiota}{\boldsymbol{\iota}}\newcommand{\bikappa}{\boldsymbol{\kappa}}\newcommand{\bilambda}{\boldsymbol{\lambda}}\newcommand{\bimu}{\boldsymbol{\mu}}\newcommand{\binu}{\boldsymbol{\nu}}\newcommand{\bixi}{\boldsymbol{\xi}}\newcommand{\biomicron}{\boldsymbol{\micron}}\newcommand{\bipi}{\boldsymbol{\pi}}\newcommand{\birho}{\boldsymbol{\rho}}\newcommand{\bisigma}{\boldsymbol{\sigma}}\newcommand{\bitau}{\boldsymbol{\tau}}\newcommand{\biupsilon}{\boldsymbol{\upsilon}}\newcommand{\biphi}{\boldsymbol{\phi}}\newcommand{\bichi}{\boldsymbol{\chi}}\newcommand{\bipsi}{\boldsymbol{\psi}}\newcommand{\biomega}{\boldsymbol{\omega}}t = 6$ seconds was set based on the participant's speed and presumed heading, and in center-to-center collision trials this was the collision point.

In center-to-center collision trials, the initial distance between the pedestrian and the participant, $\newcommand{\bialpha}{\boldsymbol{\alpha}}\newcommand{\bibeta}{\boldsymbol{\beta}}\newcommand{\bigamma}{\boldsymbol{\gamma}}\newcommand{\bidelta}{\boldsymbol{\delta}}\newcommand{\bivarepsilon}{\boldsymbol{\varepsilon}}\newcommand{\bizeta}{\boldsymbol{\zeta}}\newcommand{\bieta}{\boldsymbol{\eta}}\newcommand{\bitheta}{\boldsymbol{\theta}}\newcommand{\biiota}{\boldsymbol{\iota}}\newcommand{\bikappa}{\boldsymbol{\kappa}}\newcommand{\bilambda}{\boldsymbol{\lambda}}\newcommand{\bimu}{\boldsymbol{\mu}}\newcommand{\binu}{\boldsymbol{\nu}}\newcommand{\bixi}{\boldsymbol{\xi}}\newcommand{\biomicron}{\boldsymbol{\micron}}\newcommand{\bipi}{\boldsymbol{\pi}}\newcommand{\birho}{\boldsymbol{\rho}}\newcommand{\bisigma}{\boldsymbol{\sigma}}\newcommand{\bitau}{\boldsymbol{\tau}}\newcommand{\biupsilon}{\boldsymbol{\upsilon}}\newcommand{\biphi}{\boldsymbol{\phi}}\newcommand{\bichi}{\boldsymbol{\chi}}\newcommand{\bipsi}{\boldsymbol{\psi}}\newcommand{\biomega}{\boldsymbol{\omega}}{d_{t = 0}}$, was 19.4 m. The pedestrian's initial bearing, $\newcommand{\bialpha}{\boldsymbol{\alpha}}\newcommand{\bibeta}{\boldsymbol{\beta}}\newcommand{\bigamma}{\boldsymbol{\gamma}}\newcommand{\bidelta}{\boldsymbol{\delta}}\newcommand{\bivarepsilon}{\boldsymbol{\varepsilon}}\newcommand{\bizeta}{\boldsymbol{\zeta}}\newcommand{\bieta}{\boldsymbol{\eta}}\newcommand{\bitheta}{\boldsymbol{\theta}}\newcommand{\biiota}{\boldsymbol{\iota}}\newcommand{\bikappa}{\boldsymbol{\kappa}}\newcommand{\bilambda}{\boldsymbol{\lambda}}\newcommand{\bimu}{\boldsymbol{\mu}}\newcommand{\binu}{\boldsymbol{\nu}}\newcommand{\bixi}{\boldsymbol{\xi}}\newcommand{\biomicron}{\boldsymbol{\micron}}\newcommand{\bipi}{\boldsymbol{\pi}}\newcommand{\birho}{\boldsymbol{\rho}}\newcommand{\bisigma}{\boldsymbol{\sigma}}\newcommand{\bitau}{\boldsymbol{\tau}}\newcommand{\biupsilon}{\boldsymbol{\upsilon}}\newcommand{\biphi}{\boldsymbol{\phi}}\newcommand{\bichi}{\boldsymbol{\chi}}\newcommand{\bipsi}{\boldsymbol{\psi}}\newcommand{\biomega}{\boldsymbol{\omega}}{\beta _{t = 0}}$, relative to the participant's heading was either 30° (Fig. 3a) or 10° (Fig. 3b). The former would appear outside the residual central field but within the prism-expanded field; the latter appeared within but near the boundary of the residual central field (20° in diameter) and could be seen with simulated PFL. This design allowed us to evaluate the effect of the prisms both within the residual central field and the prism-expanded field. The pedestrian appeared randomly on the right or left side and the appearances on each side were counterbalanced. Given the pedestrian's initial position and the position when $\newcommand{\bialpha}{\boldsymbol{\alpha}}\newcommand{\bibeta}{\boldsymbol{\beta}}\newcommand{\bigamma}{\boldsymbol{\gamma}}\newcommand{\bidelta}{\boldsymbol{\delta}}\newcommand{\bivarepsilon}{\boldsymbol{\varepsilon}}\newcommand{\bizeta}{\boldsymbol{\zeta}}\newcommand{\bieta}{\boldsymbol{\eta}}\newcommand{\bitheta}{\boldsymbol{\theta}}\newcommand{\biiota}{\boldsymbol{\iota}}\newcommand{\bikappa}{\boldsymbol{\kappa}}\newcommand{\bilambda}{\boldsymbol{\lambda}}\newcommand{\bimu}{\boldsymbol{\mu}}\newcommand{\binu}{\boldsymbol{\nu}}\newcommand{\bixi}{\boldsymbol{\xi}}\newcommand{\biomicron}{\boldsymbol{\micron}}\newcommand{\bipi}{\boldsymbol{\pi}}\newcommand{\birho}{\boldsymbol{\rho}}\newcommand{\bisigma}{\boldsymbol{\sigma}}\newcommand{\bitau}{\boldsymbol{\tau}}\newcommand{\biupsilon}{\boldsymbol{\upsilon}}\newcommand{\biphi}{\boldsymbol{\phi}}\newcommand{\bichi}{\boldsymbol{\chi}}\newcommand{\bipsi}{\boldsymbol{\psi}}\newcommand{\biomega}{\boldsymbol{\omega}}t = 6$ seconds (the collision point), the pedestrian's heading ($\newcommand{\bialpha}{\boldsymbol{\alpha}}\newcommand{\bibeta}{\boldsymbol{\beta}}\newcommand{\bigamma}{\boldsymbol{\gamma}}\newcommand{\bidelta}{\boldsymbol{\delta}}\newcommand{\bivarepsilon}{\boldsymbol{\varepsilon}}\newcommand{\bizeta}{\boldsymbol{\zeta}}\newcommand{\bieta}{\boldsymbol{\eta}}\newcommand{\bitheta}{\boldsymbol{\theta}}\newcommand{\biiota}{\boldsymbol{\iota}}\newcommand{\bikappa}{\boldsymbol{\kappa}}\newcommand{\bilambda}{\boldsymbol{\lambda}}\newcommand{\bimu}{\boldsymbol{\mu}}\newcommand{\binu}{\boldsymbol{\nu}}\newcommand{\bixi}{\boldsymbol{\xi}}\newcommand{\biomicron}{\boldsymbol{\micron}}\newcommand{\bipi}{\boldsymbol{\pi}}\newcommand{\birho}{\boldsymbol{\rho}}\newcommand{\bisigma}{\boldsymbol{\sigma}}\newcommand{\bitau}{\boldsymbol{\tau}}\newcommand{\biupsilon}{\boldsymbol{\upsilon}}\newcommand{\biphi}{\boldsymbol{\phi}}\newcommand{\bichi}{\boldsymbol{\chi}}\newcommand{\bipsi}{\boldsymbol{\psi}}\newcommand{\biomega}{\boldsymbol{\omega}}{\rm{\upalpha }}$) and speed were calculated for each trial.

We also included conditions of corridor/sidewalk-like encounters, in which the heading directions of the participant and pedestrian were parallel and toward each other; the participant and pedestrian approached each other on parallel paths with a lateral displacement (Fig. 3c). As their paths were not crossing, the path crossing distance, $\newcommand{\bialpha}{\boldsymbol{\alpha}}\newcommand{\bibeta}{\boldsymbol{\beta}}\newcommand{\bigamma}{\boldsymbol{\gamma}}\newcommand{\bidelta}{\boldsymbol{\delta}}\newcommand{\bivarepsilon}{\boldsymbol{\varepsilon}}\newcommand{\bizeta}{\boldsymbol{\zeta}}\newcommand{\bieta}{\boldsymbol{\eta}}\newcommand{\bitheta}{\boldsymbol{\theta}}\newcommand{\biiota}{\boldsymbol{\iota}}\newcommand{\bikappa}{\boldsymbol{\kappa}}\newcommand{\bilambda}{\boldsymbol{\lambda}}\newcommand{\bimu}{\boldsymbol{\mu}}\newcommand{\binu}{\boldsymbol{\nu}}\newcommand{\bixi}{\boldsymbol{\xi}}\newcommand{\biomicron}{\boldsymbol{\micron}}\newcommand{\bipi}{\boldsymbol{\pi}}\newcommand{\birho}{\boldsymbol{\rho}}\newcommand{\bisigma}{\boldsymbol{\sigma}}\newcommand{\bitau}{\boldsymbol{\tau}}\newcommand{\biupsilon}{\boldsymbol{\upsilon}}\newcommand{\biphi}{\boldsymbol{\phi}}\newcommand{\bichi}{\boldsymbol{\chi}}\newcommand{\bipsi}{\boldsymbol{\psi}}\newcommand{\biomega}{\boldsymbol{\omega}}{d_{pc}}$, was recorded as ∞. Two such conditions were tested: the pedestrian could initially appear at the bearing of 5° or 2.5° (both within the residual central field), and it passed the participant laterally (1.69 m away from the participant for both 5° and 2.5°) after 6 seconds.

To evaluate whether participants could distinguish impending collisions from noncollisions, trials with near-collision and noncollision were included by changing the path crossing distances ($\newcommand{\bialpha}{\boldsymbol{\alpha}}\newcommand{\bibeta}{\boldsymbol{\beta}}\newcommand{\bigamma}{\boldsymbol{\gamma}}\newcommand{\bidelta}{\boldsymbol{\delta}}\newcommand{\bivarepsilon}{\boldsymbol{\varepsilon}}\newcommand{\bizeta}{\boldsymbol{\zeta}}\newcommand{\bieta}{\boldsymbol{\eta}}\newcommand{\bitheta}{\boldsymbol{\theta}}\newcommand{\biiota}{\boldsymbol{\iota}}\newcommand{\bikappa}{\boldsymbol{\kappa}}\newcommand{\bilambda}{\boldsymbol{\lambda}}\newcommand{\bimu}{\boldsymbol{\mu}}\newcommand{\binu}{\boldsymbol{\nu}}\newcommand{\bixi}{\boldsymbol{\xi}}\newcommand{\biomicron}{\boldsymbol{\micron}}\newcommand{\bipi}{\boldsymbol{\pi}}\newcommand{\birho}{\boldsymbol{\rho}}\newcommand{\bisigma}{\boldsymbol{\sigma}}\newcommand{\bitau}{\boldsymbol{\tau}}\newcommand{\biupsilon}{\boldsymbol{\upsilon}}\newcommand{\biphi}{\boldsymbol{\phi}}\newcommand{\bichi}{\boldsymbol{\chi}}\newcommand{\bipsi}{\boldsymbol{\psi}}\newcommand{\biomega}{\boldsymbol{\omega}}{d_{pc}}$) while maintaining the pedestrian's heading direction ($\newcommand{\bialpha}{\boldsymbol{\alpha}}\newcommand{\bibeta}{\boldsymbol{\beta}}\newcommand{\bigamma}{\boldsymbol{\gamma}}\newcommand{\bidelta}{\boldsymbol{\delta}}\newcommand{\bivarepsilon}{\boldsymbol{\varepsilon}}\newcommand{\bizeta}{\boldsymbol{\zeta}}\newcommand{\bieta}{\boldsymbol{\eta}}\newcommand{\bitheta}{\boldsymbol{\theta}}\newcommand{\biiota}{\boldsymbol{\iota}}\newcommand{\bikappa}{\boldsymbol{\kappa}}\newcommand{\bilambda}{\boldsymbol{\lambda}}\newcommand{\bimu}{\boldsymbol{\mu}}\newcommand{\binu}{\boldsymbol{\nu}}\newcommand{\bixi}{\boldsymbol{\xi}}\newcommand{\biomicron}{\boldsymbol{\micron}}\newcommand{\bipi}{\boldsymbol{\pi}}\newcommand{\birho}{\boldsymbol{\rho}}\newcommand{\bisigma}{\boldsymbol{\sigma}}\newcommand{\bitau}{\boldsymbol{\tau}}\newcommand{\biupsilon}{\boldsymbol{\upsilon}}\newcommand{\biphi}{\boldsymbol{\phi}}\newcommand{\bichi}{\boldsymbol{\chi}}\newcommand{\bipsi}{\boldsymbol{\psi}}\newcommand{\biomega}{\boldsymbol{\omega}}\alpha$). When $\newcommand{\bialpha}{\boldsymbol{\alpha}}\newcommand{\bibeta}{\boldsymbol{\beta}}\newcommand{\bigamma}{\boldsymbol{\gamma}}\newcommand{\bidelta}{\boldsymbol{\delta}}\newcommand{\bivarepsilon}{\boldsymbol{\varepsilon}}\newcommand{\bizeta}{\boldsymbol{\zeta}}\newcommand{\bieta}{\boldsymbol{\eta}}\newcommand{\bitheta}{\boldsymbol{\theta}}\newcommand{\biiota}{\boldsymbol{\iota}}\newcommand{\bikappa}{\boldsymbol{\kappa}}\newcommand{\bilambda}{\boldsymbol{\lambda}}\newcommand{\bimu}{\boldsymbol{\mu}}\newcommand{\binu}{\boldsymbol{\nu}}\newcommand{\bixi}{\boldsymbol{\xi}}\newcommand{\biomicron}{\boldsymbol{\micron}}\newcommand{\bipi}{\boldsymbol{\pi}}\newcommand{\birho}{\boldsymbol{\rho}}\newcommand{\bisigma}{\boldsymbol{\sigma}}\newcommand{\bitau}{\boldsymbol{\tau}}\newcommand{\biupsilon}{\boldsymbol{\upsilon}}\newcommand{\biphi}{\boldsymbol{\phi}}\newcommand{\bichi}{\boldsymbol{\chi}}\newcommand{\bipsi}{\boldsymbol{\psi}}\newcommand{\biomega}{\boldsymbol{\omega}}{d_{pc}}$ > 0, the pedestrian passed in front of the participant (Fig. 3d). When $\newcommand{\bialpha}{\boldsymbol{\alpha}}\newcommand{\bibeta}{\boldsymbol{\beta}}\newcommand{\bigamma}{\boldsymbol{\gamma}}\newcommand{\bidelta}{\boldsymbol{\delta}}\newcommand{\bivarepsilon}{\boldsymbol{\varepsilon}}\newcommand{\bizeta}{\boldsymbol{\zeta}}\newcommand{\bieta}{\boldsymbol{\eta}}\newcommand{\bitheta}{\boldsymbol{\theta}}\newcommand{\biiota}{\boldsymbol{\iota}}\newcommand{\bikappa}{\boldsymbol{\kappa}}\newcommand{\bilambda}{\boldsymbol{\lambda}}\newcommand{\bimu}{\boldsymbol{\mu}}\newcommand{\binu}{\boldsymbol{\nu}}\newcommand{\bixi}{\boldsymbol{\xi}}\newcommand{\biomicron}{\boldsymbol{\micron}}\newcommand{\bipi}{\boldsymbol{\pi}}\newcommand{\birho}{\boldsymbol{\rho}}\newcommand{\bisigma}{\boldsymbol{\sigma}}\newcommand{\bitau}{\boldsymbol{\tau}}\newcommand{\biupsilon}{\boldsymbol{\upsilon}}\newcommand{\biphi}{\boldsymbol{\phi}}\newcommand{\bichi}{\boldsymbol{\chi}}\newcommand{\bipsi}{\boldsymbol{\psi}}\newcommand{\biomega}{\boldsymbol{\omega}}{d_{pc}} \lt 0$, the pedestrian passed behind the participant (Fig. 3e). For each initial bearing, $\newcommand{\bialpha}{\boldsymbol{\alpha}}\newcommand{\bibeta}{\boldsymbol{\beta}}\newcommand{\bigamma}{\boldsymbol{\gamma}}\newcommand{\bidelta}{\boldsymbol{\delta}}\newcommand{\bivarepsilon}{\boldsymbol{\varepsilon}}\newcommand{\bizeta}{\boldsymbol{\zeta}}\newcommand{\bieta}{\boldsymbol{\eta}}\newcommand{\bitheta}{\boldsymbol{\theta}}\newcommand{\biiota}{\boldsymbol{\iota}}\newcommand{\bikappa}{\boldsymbol{\kappa}}\newcommand{\bilambda}{\boldsymbol{\lambda}}\newcommand{\bimu}{\boldsymbol{\mu}}\newcommand{\binu}{\boldsymbol{\nu}}\newcommand{\bixi}{\boldsymbol{\xi}}\newcommand{\biomicron}{\boldsymbol{\micron}}\newcommand{\bipi}{\boldsymbol{\pi}}\newcommand{\birho}{\boldsymbol{\rho}}\newcommand{\bisigma}{\boldsymbol{\sigma}}\newcommand{\bitau}{\boldsymbol{\tau}}\newcommand{\biupsilon}{\boldsymbol{\upsilon}}\newcommand{\biphi}{\boldsymbol{\phi}}\newcommand{\bichi}{\boldsymbol{\chi}}\newcommand{\bipsi}{\boldsymbol{\psi}}\newcommand{\biomega}{\boldsymbol{\omega}}{\beta _{t = 0}}$, three nonzero $\newcommand{\bialpha}{\boldsymbol{\alpha}}\newcommand{\bibeta}{\boldsymbol{\beta}}\newcommand{\bigamma}{\boldsymbol{\gamma}}\newcommand{\bidelta}{\boldsymbol{\delta}}\newcommand{\bivarepsilon}{\boldsymbol{\varepsilon}}\newcommand{\bizeta}{\boldsymbol{\zeta}}\newcommand{\bieta}{\boldsymbol{\eta}}\newcommand{\bitheta}{\boldsymbol{\theta}}\newcommand{\biiota}{\boldsymbol{\iota}}\newcommand{\bikappa}{\boldsymbol{\kappa}}\newcommand{\bilambda}{\boldsymbol{\lambda}}\newcommand{\bimu}{\boldsymbol{\mu}}\newcommand{\binu}{\boldsymbol{\nu}}\newcommand{\bixi}{\boldsymbol{\xi}}\newcommand{\biomicron}{\boldsymbol{\micron}}\newcommand{\bipi}{\boldsymbol{\pi}}\newcommand{\birho}{\boldsymbol{\rho}}\newcommand{\bisigma}{\boldsymbol{\sigma}}\newcommand{\bitau}{\boldsymbol{\tau}}\newcommand{\biupsilon}{\boldsymbol{\upsilon}}\newcommand{\biphi}{\boldsymbol{\phi}}\newcommand{\bichi}{\boldsymbol{\chi}}\newcommand{\bipsi}{\boldsymbol{\psi}}\newcommand{\biomega}{\boldsymbol{\omega}}{d_{pc}}$, −2 m, +2 m, and +12 m, were tested. These distances were selected based on a preliminary study in the same environment^29^: we found for pedestrians passing in front of the normally sighted subjects that the threshold path crossing distance for perceived collision (50% trials decided as collision) was +6 m, and for pedestrians passing behind, the threshold was about −2 m. Since the closest distance between the participant and the pedestrian was smaller than 1 m for $\newcommand{\bialpha}{\boldsymbol{\alpha}}\newcommand{\bibeta}{\boldsymbol{\beta}}\newcommand{\bigamma}{\boldsymbol{\gamma}}\newcommand{\bidelta}{\boldsymbol{\delta}}\newcommand{\bivarepsilon}{\boldsymbol{\varepsilon}}\newcommand{\bizeta}{\boldsymbol{\zeta}}\newcommand{\bieta}{\boldsymbol{\eta}}\newcommand{\bitheta}{\boldsymbol{\theta}}\newcommand{\biiota}{\boldsymbol{\iota}}\newcommand{\bikappa}{\boldsymbol{\kappa}}\newcommand{\bilambda}{\boldsymbol{\lambda}}\newcommand{\bimu}{\boldsymbol{\mu}}\newcommand{\binu}{\boldsymbol{\nu}}\newcommand{\bixi}{\boldsymbol{\xi}}\newcommand{\biomicron}{\boldsymbol{\micron}}\newcommand{\bipi}{\boldsymbol{\pi}}\newcommand{\birho}{\boldsymbol{\rho}}\newcommand{\bisigma}{\boldsymbol{\sigma}}\newcommand{\bitau}{\boldsymbol{\tau}}\newcommand{\biupsilon}{\boldsymbol{\upsilon}}\newcommand{\biphi}{\boldsymbol{\phi}}\newcommand{\bichi}{\boldsymbol{\chi}}\newcommand{\bipsi}{\boldsymbol{\psi}}\newcommand{\biomega}{\boldsymbol{\omega}}{d_{pc}} = - 2$ m and +2 m, these conditions were counted as near-collisions (thus *collision* response was counted as correct), while the conditions with $\newcommand{\bialpha}{\boldsymbol{\alpha}}\newcommand{\bibeta}{\boldsymbol{\beta}}\newcommand{\bigamma}{\boldsymbol{\gamma}}\newcommand{\bidelta}{\boldsymbol{\delta}}\newcommand{\bivarepsilon}{\boldsymbol{\varepsilon}}\newcommand{\bizeta}{\boldsymbol{\zeta}}\newcommand{\bieta}{\boldsymbol{\eta}}\newcommand{\bitheta}{\boldsymbol{\theta}}\newcommand{\biiota}{\boldsymbol{\iota}}\newcommand{\bikappa}{\boldsymbol{\kappa}}\newcommand{\bilambda}{\boldsymbol{\lambda}}\newcommand{\bimu}{\boldsymbol{\mu}}\newcommand{\binu}{\boldsymbol{\nu}}\newcommand{\bixi}{\boldsymbol{\xi}}\newcommand{\biomicron}{\boldsymbol{\micron}}\newcommand{\bipi}{\boldsymbol{\pi}}\newcommand{\birho}{\boldsymbol{\rho}}\newcommand{\bisigma}{\boldsymbol{\sigma}}\newcommand{\bitau}{\boldsymbol{\tau}}\newcommand{\biupsilon}{\boldsymbol{\upsilon}}\newcommand{\biphi}{\boldsymbol{\phi}}\newcommand{\bichi}{\boldsymbol{\chi}}\newcommand{\bipsi}{\boldsymbol{\psi}}\newcommand{\biomega}{\boldsymbol{\omega}}{d_{pc}} = + 12$ m were noncollisions. In addition, the conditions with the participant and pedestrian on parallel paths (Fig. 3c) were noncollisions since the closest distance between their centers (1.69 m for both 5° and 2.5°) was much larger than the 0.6 m body width.

The 20 trials/pedestrians in each scenario included 10 trials on the right and 10 trials on the left. Two out of the 10 trials were parallel noncollision conditions; four trials were with $\newcommand{\bialpha}{\boldsymbol{\alpha}}\newcommand{\bibeta}{\boldsymbol{\beta}}\newcommand{\bigamma}{\boldsymbol{\gamma}}\newcommand{\bidelta}{\boldsymbol{\delta}}\newcommand{\bivarepsilon}{\boldsymbol{\varepsilon}}\newcommand{\bizeta}{\boldsymbol{\zeta}}\newcommand{\bieta}{\boldsymbol{\eta}}\newcommand{\bitheta}{\boldsymbol{\theta}}\newcommand{\biiota}{\boldsymbol{\iota}}\newcommand{\bikappa}{\boldsymbol{\kappa}}\newcommand{\bilambda}{\boldsymbol{\lambda}}\newcommand{\bimu}{\boldsymbol{\mu}}\newcommand{\binu}{\boldsymbol{\nu}}\newcommand{\bixi}{\boldsymbol{\xi}}\newcommand{\biomicron}{\boldsymbol{\micron}}\newcommand{\bipi}{\boldsymbol{\pi}}\newcommand{\birho}{\boldsymbol{\rho}}\newcommand{\bisigma}{\boldsymbol{\sigma}}\newcommand{\bitau}{\boldsymbol{\tau}}\newcommand{\biupsilon}{\boldsymbol{\upsilon}}\newcommand{\biphi}{\boldsymbol{\phi}}\newcommand{\bichi}{\boldsymbol{\chi}}\newcommand{\bipsi}{\boldsymbol{\psi}}\newcommand{\biomega}{\boldsymbol{\omega}}{\beta _{t = 0}} = 10^{\circ}$, with $\newcommand{\bialpha}{\boldsymbol{\alpha}}\newcommand{\bibeta}{\boldsymbol{\beta}}\newcommand{\bigamma}{\boldsymbol{\gamma}}\newcommand{\bidelta}{\boldsymbol{\delta}}\newcommand{\bivarepsilon}{\boldsymbol{\varepsilon}}\newcommand{\bizeta}{\boldsymbol{\zeta}}\newcommand{\bieta}{\boldsymbol{\eta}}\newcommand{\bitheta}{\boldsymbol{\theta}}\newcommand{\biiota}{\boldsymbol{\iota}}\newcommand{\bikappa}{\boldsymbol{\kappa}}\newcommand{\bilambda}{\boldsymbol{\lambda}}\newcommand{\bimu}{\boldsymbol{\mu}}\newcommand{\binu}{\boldsymbol{\nu}}\newcommand{\bixi}{\boldsymbol{\xi}}\newcommand{\biomicron}{\boldsymbol{\micron}}\newcommand{\bipi}{\boldsymbol{\pi}}\newcommand{\birho}{\boldsymbol{\rho}}\newcommand{\bisigma}{\boldsymbol{\sigma}}\newcommand{\bitau}{\boldsymbol{\tau}}\newcommand{\biupsilon}{\boldsymbol{\upsilon}}\newcommand{\biphi}{\boldsymbol{\phi}}\newcommand{\bichi}{\boldsymbol{\chi}}\newcommand{\bipsi}{\boldsymbol{\psi}}\newcommand{\biomega}{\boldsymbol{\omega}}{d_{pc}} = - 2$ m, 0, +2 m, and +12 m, respectively; and the other four trials were with $\newcommand{\bialpha}{\boldsymbol{\alpha}}\newcommand{\bibeta}{\boldsymbol{\beta}}\newcommand{\bigamma}{\boldsymbol{\gamma}}\newcommand{\bidelta}{\boldsymbol{\delta}}\newcommand{\bivarepsilon}{\boldsymbol{\varepsilon}}\newcommand{\bizeta}{\boldsymbol{\zeta}}\newcommand{\bieta}{\boldsymbol{\eta}}\newcommand{\bitheta}{\boldsymbol{\theta}}\newcommand{\biiota}{\boldsymbol{\iota}}\newcommand{\bikappa}{\boldsymbol{\kappa}}\newcommand{\bilambda}{\boldsymbol{\lambda}}\newcommand{\bimu}{\boldsymbol{\mu}}\newcommand{\binu}{\boldsymbol{\nu}}\newcommand{\bixi}{\boldsymbol{\xi}}\newcommand{\biomicron}{\boldsymbol{\micron}}\newcommand{\bipi}{\boldsymbol{\pi}}\newcommand{\birho}{\boldsymbol{\rho}}\newcommand{\bisigma}{\boldsymbol{\sigma}}\newcommand{\bitau}{\boldsymbol{\tau}}\newcommand{\biupsilon}{\boldsymbol{\upsilon}}\newcommand{\biphi}{\boldsymbol{\phi}}\newcommand{\bichi}{\boldsymbol{\chi}}\newcommand{\bipsi}{\boldsymbol{\psi}}\newcommand{\biomega}{\boldsymbol{\omega}}{\beta _{t = 0}} = 30^{\circ}$, with the same four $\newcommand{\bialpha}{\boldsymbol{\alpha}}\newcommand{\bibeta}{\boldsymbol{\beta}}\newcommand{\bigamma}{\boldsymbol{\gamma}}\newcommand{\bidelta}{\boldsymbol{\delta}}\newcommand{\bivarepsilon}{\boldsymbol{\varepsilon}}\newcommand{\bizeta}{\boldsymbol{\zeta}}\newcommand{\bieta}{\boldsymbol{\eta}}\newcommand{\bitheta}{\boldsymbol{\theta}}\newcommand{\biiota}{\boldsymbol{\iota}}\newcommand{\bikappa}{\boldsymbol{\kappa}}\newcommand{\bilambda}{\boldsymbol{\lambda}}\newcommand{\bimu}{\boldsymbol{\mu}}\newcommand{\binu}{\boldsymbol{\nu}}\newcommand{\bixi}{\boldsymbol{\xi}}\newcommand{\biomicron}{\boldsymbol{\micron}}\newcommand{\bipi}{\boldsymbol{\pi}}\newcommand{\birho}{\boldsymbol{\rho}}\newcommand{\bisigma}{\boldsymbol{\sigma}}\newcommand{\bitau}{\boldsymbol{\tau}}\newcommand{\biupsilon}{\boldsymbol{\upsilon}}\newcommand{\biphi}{\boldsymbol{\phi}}\newcommand{\bichi}{\boldsymbol{\chi}}\newcommand{\bipsi}{\boldsymbol{\psi}}\newcommand{\biomega}{\boldsymbol{\omega}}{d_{pc}}$ values. The order of the trials was randomized in each scenario to counterbalance possibly confounding effects of various background contents and to minimize potential effects of learning within the scenario.

#### Participants' Task

Besides maintaining the path by steering to align the basketball and string, participants were instructed to first press the horn as quickly as possible when they detected the pedestrian, and a second time when they decided whether the pedestrian was on a collision course with their body or not. For the collision judgment task, if they decided that the pedestrian would not collide, they pressed the horn once; if they believed the pedestrian would collide, they pressed the horn twice. This allowed us to collect the participants' pedestrian detection and collision judgment, as well as the response time (RT) for both responses.

### Part II. Goggles to Simulate PFL and Test Prisms

#### 

**Figure 4:**
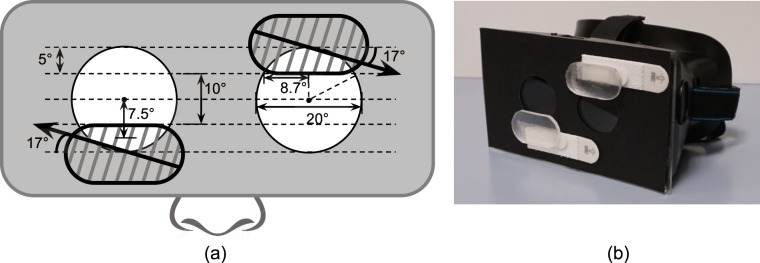
Goggles for simulating PFL. (a) Front diagram of the goggles with the mask to simulate the 20° diameter residual central field and the oblique Fresnel prisms. The arrows indicate the direction from the prism apex to base, thus the prisms are placed base-out and base-up in front of the right eye, and base-out and base-down in front of the left eye. (b) Picture of the goggles with the prisms attached.

To conduct an evaluation of the test environment and the impact of the field of view expanding prisms, we developed goggles to simulate PFL in normally sighted subjects. A 20° diameter binocular residual central field was simulated using open apertures on head-mounted opaque goggles (Fig. 4). The front cover of the goggles was replaced with a clear acrylic surface (Fig. 4b). A customized aperture mask adjusted for each individual subject's parameters (including interpupillary distance, back vertex distance, and convergence angle when viewing the driving simulator screens) was attached to the acrylic surface to restrict the peripheral field (Fig. 4). The goggles with about 100 mm back vertex distance (between the back of the acrylic surface to the front of the cornea) were used to allow larger apertures for a 20° diameter field. The larger apertures avoided diffraction caused by small apertures that were required with small vertex distance.^30^

##### Prism Placement on Glasses

We first briefly describe a design for prism placement on actual lenses of glasses for PFL (see Supplementary Appendix “Details of prism positioning” for more details), and then describe its adaptation to our goggles simulating PFL. The prisms are intended to only cover the upper and lower residual paracentral field, leaving the central vertical 10° (in diameter) clear to avoid central double vision when at primary gaze. The design was derived from the analyses presented in Apfelbaum and Peli^2^ and Peli and Jung.^19^ Such design spares the central field from incorrectly perceiving the direction of the walking target, avoids misguided locomotion,^31–33^ and prevents losing central binocular fusion with consequent central double vision. Central double vision has been identified as a limiting side effect of prior field of view expansion prism designs.^2,34^ This 10° (in diameter) of vertical central field clear of prisms was designed for patients with 20° diameter residual central fields.

Oblique 57Δ Fresnel prisms were embedded into the upper and lower peripheral portions of the spectacle lenses, as done with the commercially available peripheral prisms for HH, but with narrower interprism separation for a smaller field size and with oblique tilt (see Supplementary Appendix “Details of prism positioning” for more details). The upper or lower prisms were placed only in front of one eye, with the fellow eye maintaining an un-shifted paracentral view that was blocked by the prism.^22^ Such design enables *biocular multiplexing* in field of view expansion.^21^ Unlike the standard fitting for HH, where both upper and lower segments are placed on the same lens with both bases in the same direction, here we placed one on each lens. Both prisms were placed base-out, with the right lens prism expanding the right-eye field of view to the right and the left lens prism expanding the left-eye field of view to the left.

##### Prisms for Simulated PFL Using Goggles

The oblique 57Δ Fresnel prisms were placed on the surface of the goggles to cover the upper and lower 5° of the aperture, leaving the central vertical 10° diameter clear (Fig. 4a). The widest horizontal field of view within the prism in the goggles (and in the glasses, without vertical eye movements) is about $\newcommand{\bialpha}{\boldsymbol{\alpha}}\newcommand{\bibeta}{\boldsymbol{\beta}}\newcommand{\bigamma}{\boldsymbol{\gamma}}\newcommand{\bidelta}{\boldsymbol{\delta}}\newcommand{\bivarepsilon}{\boldsymbol{\varepsilon}}\newcommand{\bizeta}{\boldsymbol{\zeta}}\newcommand{\bieta}{\boldsymbol{\eta}}\newcommand{\bitheta}{\boldsymbol{\theta}}\newcommand{\biiota}{\boldsymbol{\iota}}\newcommand{\bikappa}{\boldsymbol{\kappa}}\newcommand{\bilambda}{\boldsymbol{\lambda}}\newcommand{\bimu}{\boldsymbol{\mu}}\newcommand{\binu}{\boldsymbol{\nu}}\newcommand{\bixi}{\boldsymbol{\xi}}\newcommand{\biomicron}{\boldsymbol{\micron}}\newcommand{\bipi}{\boldsymbol{\pi}}\newcommand{\birho}{\boldsymbol{\rho}}\newcommand{\bisigma}{\boldsymbol{\sigma}}\newcommand{\bitau}{\boldsymbol{\tau}}\newcommand{\biupsilon}{\boldsymbol{\upsilon}}\newcommand{\biphi}{\boldsymbol{\phi}}\newcommand{\bichi}{\boldsymbol{\chi}}\newcommand{\bipsi}{\boldsymbol{\psi}}\newcommand{\biomega}{\boldsymbol{\omega}}2 \times 8.7\circ = 17.4^{\circ}$ (Fig. 4a). Furthermore, since the upper and lower prism-expanded fields were vertically centered at ±7.5° from the primary gaze ($\newcommand{\bialpha}{\boldsymbol{\alpha}}\newcommand{\bibeta}{\boldsymbol{\beta}}\newcommand{\bigamma}{\boldsymbol{\gamma}}\newcommand{\bidelta}{\boldsymbol{\delta}}\newcommand{\bivarepsilon}{\boldsymbol{\varepsilon}}\newcommand{\bizeta}{\boldsymbol{\zeta}}\newcommand{\bieta}{\boldsymbol{\eta}}\newcommand{\bitheta}{\boldsymbol{\theta}}\newcommand{\biiota}{\boldsymbol{\iota}}\newcommand{\bikappa}{\boldsymbol{\kappa}}\newcommand{\bilambda}{\boldsymbol{\lambda}}\newcommand{\bimu}{\boldsymbol{\mu}}\newcommand{\binu}{\boldsymbol{\nu}}\newcommand{\bixi}{\boldsymbol{\xi}}\newcommand{\biomicron}{\boldsymbol{\micron}}\newcommand{\bipi}{\boldsymbol{\pi}}\newcommand{\birho}{\boldsymbol{\rho}}\newcommand{\bisigma}{\boldsymbol{\sigma}}\newcommand{\bitau}{\boldsymbol{\tau}}\newcommand{\biupsilon}{\boldsymbol{\upsilon}}\newcommand{\biphi}{\boldsymbol{\phi}}\newcommand{\bichi}{\boldsymbol{\chi}}\newcommand{\bipsi}{\boldsymbol{\psi}}\newcommand{\biomega}{\boldsymbol{\omega}}0^{\circ}$ eccentricity), a ±7.5° vertical prism shift was required to bring the center of the prism-expanded fields toward eye level. With the prism-expanded fields located at eye level, the upper and lower prisms can better contribute to pedestrian detection. The tilted angle of the prism apex-base axis was calculated as 17° (Fig. 4a), which resulted in a slight reduction of horizontal effective angle deviation at the primary position of gaze (see details in Supplementary Appendix “Details of prism positioning”).

**Figure 5:**
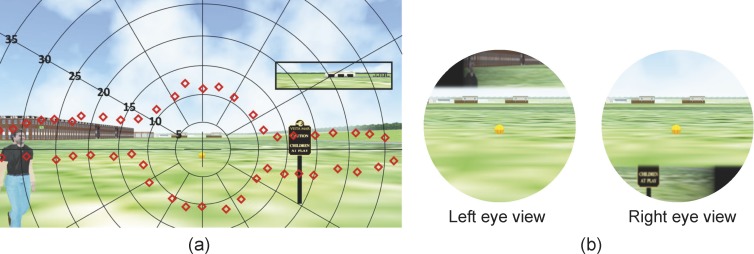
Illustration of the simulated PFL and prism effects. (a) Binocular perimetry through the simulated PFL using goggles with the prisms. A scene from the virtual scenario from the simulator front screen is overlaid by the perimetry result, which was measured with the prisms in place using a 1.5° black square target (on white screen) from 735 mm viewing distance. Note that without prisms, the expanded areas to the left and right would not be present. The measured expansion is close to the calculated theoretical values (between 11° to 39°). The black rectangular box presents the simulator rear-view mirror images, which was outside the field of simulated PFL. (b) Illustration of percepts for left and right eyes (field of view as two apertures). The vertical middle portion in both apertures is seen by both eyes and is fused, but the left and right expanded fields, as shown in (a), were each seen only through one eye. The left expanded field appears in the upper portion of the left eye view, and the right expanded field appears in the lower portion of right eye view. The far ends of expanded fields are highly compressed (note the laterally compressed head and torso of the pedestrian in the left eye's view). Areas of TIR (Supplementary Appendix “Details of prism positioning”) shown in black can be seen in the left and right ends of the left and right eyes' views, respectively. Under binocular viewing the upper and lower un-shifted fields of view will be seen by the other eye.

The two 57Δ oblique base-out prisms were placed in front of the left and right eyes, above and below primary gaze, respectively. We used a left-top and right-bottom design (Fig. 4), but the opposite configuration should be equivalent. The oblique prisms could theoretically create peripheral lateral islands between 11° and 39° of eccentricity (see Supplementary Appendix “Peripheral islands created by the prisms” for calculation). Therefore, our scripted pedestrians that appear at the 30° horizontal bearing around eye level should fall within the prism-expanded fields. This was confirmed with perimetry, as shown in Figure 5a. From the subjects' viewpoint, the expanded left/right areas outside the residual central field, seen by the left/right eye, would perceptually appear in the paracentral upper/lower visual field, respectively (Fig. 5b). The un-shifted fields of view were maintained by the other eye.

Subjects were instructed to look through the central area, and no vertical head tilt or gaze shift was required in using the device. They were explicitly instructed that objects appeared in the upper field were from the left, and objects appeared in the lower field were from the right. To familiarize the subjects with the device, they were asked to wear the goggles prisms and moved their hands in front the goggles from the far left to the right side, and to experience how, when, and where the images of their hands would appear in the upper field of view or the lower field of view, and in the central visual field.

### Part III. Evaluating Pedestrian Detection and Collision Judgment

#### Procedure

Nine normally sighted subjects with normal or corrected to normal visual acuity (average age 27, three females) participated in the experiment. All subjects were fitted with the customized goggles simulating 20° diameter binocular residual central field and then shown the field of view expanding prisms. The perceptual effects of the prisms were explicitly explained. All subjects were tested under three viewing conditions: normal vision (NV) without the goggles, simulated PFL with the goggles (PFL), and simulated PFL with the field of view expanding prisms placed on the goggles (PFL+PR).

The subjects were given ample time (20 to 30 minutes including the goggles and prisms familiarization) to practice the experimental tasks in training scenarios that contained the same string alignment, pedestrian detection, and collision decision tasks, but did not contain the exact pedestrian trials that were tested in the study. The NV condition was practiced first, followed by the PFL and PFL+PR conditions. The experiment comprised six scenarios (each with 20 trials), two scenarios for each viewing condition. The order of the scenarios and the viewing conditions were randomized for each subject.

To determine the face validity of simulated PFL in the virtual environment, eight patients with real PFL (average age 44, five females) were also recruited. Their visual acuity varied from logMAR of 0.02 to 0.82 (median logMAR, 0.27; interquartile range [IQR], 0.19–0.34). The diameter of the patient's binocular residual central field ranged from 16° to 56° (median, 21.5°; IQR, 19°–37.5°). They all used their best-corrected vision with no prismatic lenses, and each completed three scenarios (60 trials). Written informed consent under an experimental protocol approved by the Massachusetts Eye and Ear Human Studies Committee was obtained from all participants prior to the experiment.

#### Data Analysis

The time between the pedestrian appearance and the participant's first horn press was recorded as the RT for detection (detection RT), and the time between the first and the second set of horn presses (collision judgment) was recorded as the decision RT. Data were first grouped based on whether the pedestrian's initial bearing ($\newcommand{\bialpha}{\boldsymbol{\alpha}}\newcommand{\bibeta}{\boldsymbol{\beta}}\newcommand{\bigamma}{\boldsymbol{\gamma}}\newcommand{\bidelta}{\boldsymbol{\delta}}\newcommand{\bivarepsilon}{\boldsymbol{\varepsilon}}\newcommand{\bizeta}{\boldsymbol{\zeta}}\newcommand{\bieta}{\boldsymbol{\eta}}\newcommand{\bitheta}{\boldsymbol{\theta}}\newcommand{\biiota}{\boldsymbol{\iota}}\newcommand{\bikappa}{\boldsymbol{\kappa}}\newcommand{\bilambda}{\boldsymbol{\lambda}}\newcommand{\bimu}{\boldsymbol{\mu}}\newcommand{\binu}{\boldsymbol{\nu}}\newcommand{\bixi}{\boldsymbol{\xi}}\newcommand{\biomicron}{\boldsymbol{\micron}}\newcommand{\bipi}{\boldsymbol{\pi}}\newcommand{\birho}{\boldsymbol{\rho}}\newcommand{\bisigma}{\boldsymbol{\sigma}}\newcommand{\bitau}{\boldsymbol{\tau}}\newcommand{\biupsilon}{\boldsymbol{\upsilon}}\newcommand{\biphi}{\boldsymbol{\phi}}\newcommand{\bichi}{\boldsymbol{\chi}}\newcommand{\bipsi}{\boldsymbol{\psi}}\newcommand{\biomega}{\boldsymbol{\omega}}{\beta _{t = 0}}$) was larger than 10°, to compare performance within the residual central ($\newcommand{\bialpha}{\boldsymbol{\alpha}}\newcommand{\bibeta}{\boldsymbol{\beta}}\newcommand{\bigamma}{\boldsymbol{\gamma}}\newcommand{\bidelta}{\boldsymbol{\delta}}\newcommand{\bivarepsilon}{\boldsymbol{\varepsilon}}\newcommand{\bizeta}{\boldsymbol{\zeta}}\newcommand{\bieta}{\boldsymbol{\eta}}\newcommand{\bitheta}{\boldsymbol{\theta}}\newcommand{\biiota}{\boldsymbol{\iota}}\newcommand{\bikappa}{\boldsymbol{\kappa}}\newcommand{\bilambda}{\boldsymbol{\lambda}}\newcommand{\bimu}{\boldsymbol{\mu}}\newcommand{\binu}{\boldsymbol{\nu}}\newcommand{\bixi}{\boldsymbol{\xi}}\newcommand{\biomicron}{\boldsymbol{\micron}}\newcommand{\bipi}{\boldsymbol{\pi}}\newcommand{\birho}{\boldsymbol{\rho}}\newcommand{\bisigma}{\boldsymbol{\sigma}}\newcommand{\bitau}{\boldsymbol{\tau}}\newcommand{\biupsilon}{\boldsymbol{\upsilon}}\newcommand{\biphi}{\boldsymbol{\phi}}\newcommand{\bichi}{\boldsymbol{\chi}}\newcommand{\bipsi}{\boldsymbol{\psi}}\newcommand{\biomega}{\boldsymbol{\omega}}{\beta _{t = 0}} \le 10^{\circ}$) and the prism-expanded ($\newcommand{\bialpha}{\boldsymbol{\alpha}}\newcommand{\bibeta}{\boldsymbol{\beta}}\newcommand{\bigamma}{\boldsymbol{\gamma}}\newcommand{\bidelta}{\boldsymbol{\delta}}\newcommand{\bivarepsilon}{\boldsymbol{\varepsilon}}\newcommand{\bizeta}{\boldsymbol{\zeta}}\newcommand{\bieta}{\boldsymbol{\eta}}\newcommand{\bitheta}{\boldsymbol{\theta}}\newcommand{\biiota}{\boldsymbol{\iota}}\newcommand{\bikappa}{\boldsymbol{\kappa}}\newcommand{\bilambda}{\boldsymbol{\lambda}}\newcommand{\bimu}{\boldsymbol{\mu}}\newcommand{\binu}{\boldsymbol{\nu}}\newcommand{\bixi}{\boldsymbol{\xi}}\newcommand{\biomicron}{\boldsymbol{\micron}}\newcommand{\bipi}{\boldsymbol{\pi}}\newcommand{\birho}{\boldsymbol{\rho}}\newcommand{\bisigma}{\boldsymbol{\sigma}}\newcommand{\bitau}{\boldsymbol{\tau}}\newcommand{\biupsilon}{\boldsymbol{\upsilon}}\newcommand{\biphi}{\boldsymbol{\phi}}\newcommand{\bichi}{\boldsymbol{\chi}}\newcommand{\bipsi}{\boldsymbol{\psi}}\newcommand{\biomega}{\boldsymbol{\omega}}{\beta _{t = 0}}\ \gt \ 10^{\circ}$) fields. A two-way within-subject analysis of variance (ANOVA) was conducted for the grouped data with the two independent variables—the three viewing conditions (NV, PFL, and PFL+PR) and the two field of view positions (the residual central and prism-expanded fields). For the patients, one-way within-subject ANOVAs were used to test the effect of field of view positions on detection rates, decisions, or RTs. To compare the patients' results with the simulated PFL results in normally sighted subjects, two-way between-subjects ANOVAs (participants type × field of view positions) were conducted. Furthermore, individual pedestrian conditions with various path crossing distances (collapsed over left and right pedestrians) were inspected among the normally sighted subjects using a three-way within-subject ANOVA (viewing condition × initial bearing × path crossing distance). Similarly, among the patients, two-way within-subject ANOVAs (initial bearing × path crossing distance) were conducted.

Statistics were conducted using the R package “Analysis of Factorial Experiments” (afex, https://github.com/singmann/afex). The number of degrees of freedom of the F distribution was corrected using the Greenhouse-Geisser correction^35^ to ensure the assumption of sphericity; that is, the variances of the differences between all combinations of related groups should be equal. The *P*-values in posthoc contrast tests were adjusted using the Holm-Bonferroni method^36^ for multiple comparisons.

The correlations between the subjects' behaviors (e.g., the percent of perceived collisions) and various factors (e.g., the closest distance between the pedestrian and subject) were also explored in the NV condition to determine whether subjects make comparable collision/noncollision decisions in the virtual scenarios as in real world. This was to further determine the face validity of our virtual environment.

## Results

### Pedestrians Within and Outside the Residual Central Field

**Figure 6:**
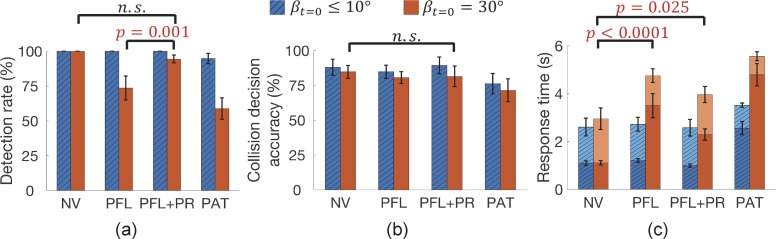
The grouped detection, decision, and response time (RT) results for the residual central field (hatched blue bars) and the prism-expanded field or the unseen field for patients (solid orange), and for the three viewing conditions or patients: NV, normal vision; PFL, simulated peripheral field loss; PFL+PR, simulated PFL with prisms; PAT, patients with PFL. (a) The detection rate. (b) The collision decision accuracy. (c) The RT for detection (bottom and darker) and decision (stacked on top and lighter color). Error bars represent standard error of the mean.

We first grouped responses based on whether the pedestrian would initially show within or outside the residual central field for both the normally sighted subjects and the patients with real PFL. When the pedestrian fell outside the residual central field, it would fall in the prism-expanded field of the normally sighted subjects and the unseen field for the patients. Among the normally sighted subjects, when the pedestrians initially appeared within the residual central field ($\newcommand{\bialpha}{\boldsymbol{\alpha}}\newcommand{\bibeta}{\boldsymbol{\beta}}\newcommand{\bigamma}{\boldsymbol{\gamma}}\newcommand{\bidelta}{\boldsymbol{\delta}}\newcommand{\bivarepsilon}{\boldsymbol{\varepsilon}}\newcommand{\bizeta}{\boldsymbol{\zeta}}\newcommand{\bieta}{\boldsymbol{\eta}}\newcommand{\bitheta}{\boldsymbol{\theta}}\newcommand{\biiota}{\boldsymbol{\iota}}\newcommand{\bikappa}{\boldsymbol{\kappa}}\newcommand{\bilambda}{\boldsymbol{\lambda}}\newcommand{\bimu}{\boldsymbol{\mu}}\newcommand{\binu}{\boldsymbol{\nu}}\newcommand{\bixi}{\boldsymbol{\xi}}\newcommand{\biomicron}{\boldsymbol{\micron}}\newcommand{\bipi}{\boldsymbol{\pi}}\newcommand{\birho}{\boldsymbol{\rho}}\newcommand{\bisigma}{\boldsymbol{\sigma}}\newcommand{\bitau}{\boldsymbol{\tau}}\newcommand{\biupsilon}{\boldsymbol{\upsilon}}\newcommand{\biphi}{\boldsymbol{\phi}}\newcommand{\bichi}{\boldsymbol{\chi}}\newcommand{\bipsi}{\boldsymbol{\psi}}\newcommand{\biomega}{\boldsymbol{\omega}}{\beta _{t = 0}} \le 10^{\circ}$), the detection rate was 100% for all the viewing conditions. For the conditions with $\newcommand{\bialpha}{\boldsymbol{\alpha}}\newcommand{\bibeta}{\boldsymbol{\beta}}\newcommand{\bigamma}{\boldsymbol{\gamma}}\newcommand{\bidelta}{\boldsymbol{\delta}}\newcommand{\bivarepsilon}{\boldsymbol{\varepsilon}}\newcommand{\bizeta}{\boldsymbol{\zeta}}\newcommand{\bieta}{\boldsymbol{\eta}}\newcommand{\bitheta}{\boldsymbol{\theta}}\newcommand{\biiota}{\boldsymbol{\iota}}\newcommand{\bikappa}{\boldsymbol{\kappa}}\newcommand{\bilambda}{\boldsymbol{\lambda}}\newcommand{\bimu}{\boldsymbol{\mu}}\newcommand{\binu}{\boldsymbol{\nu}}\newcommand{\bixi}{\boldsymbol{\xi}}\newcommand{\biomicron}{\boldsymbol{\micron}}\newcommand{\bipi}{\boldsymbol{\pi}}\newcommand{\birho}{\boldsymbol{\rho}}\newcommand{\bisigma}{\boldsymbol{\sigma}}\newcommand{\bitau}{\boldsymbol{\tau}}\newcommand{\biupsilon}{\boldsymbol{\upsilon}}\newcommand{\biphi}{\boldsymbol{\phi}}\newcommand{\bichi}{\boldsymbol{\chi}}\newcommand{\bipsi}{\boldsymbol{\psi}}\newcommand{\biomega}{\boldsymbol{\omega}}{\beta _{t = 0}} = 30^{\circ}$, the pedestrian detection rate dropped in the simulated PFL condition (74% on average) but was improved with the prisms (94%) (Fig. 6a). There was a significant interaction between the three viewing conditions (NV, PFL, and PFL+PR) and the two field of view positions (the simulated residual central field and the prism-expanded field) for the average detection rate ($\newcommand{\bialpha}{\boldsymbol{\alpha}}\newcommand{\bibeta}{\boldsymbol{\beta}}\newcommand{\bigamma}{\boldsymbol{\gamma}}\newcommand{\bidelta}{\boldsymbol{\delta}}\newcommand{\bivarepsilon}{\boldsymbol{\varepsilon}}\newcommand{\bizeta}{\boldsymbol{\zeta}}\newcommand{\bieta}{\boldsymbol{\eta}}\newcommand{\bitheta}{\boldsymbol{\theta}}\newcommand{\biiota}{\boldsymbol{\iota}}\newcommand{\bikappa}{\boldsymbol{\kappa}}\newcommand{\bilambda}{\boldsymbol{\lambda}}\newcommand{\bimu}{\boldsymbol{\mu}}\newcommand{\binu}{\boldsymbol{\nu}}\newcommand{\bixi}{\boldsymbol{\xi}}\newcommand{\biomicron}{\boldsymbol{\micron}}\newcommand{\bipi}{\boldsymbol{\pi}}\newcommand{\birho}{\boldsymbol{\rho}}\newcommand{\bisigma}{\boldsymbol{\sigma}}\newcommand{\bitau}{\boldsymbol{\tau}}\newcommand{\biupsilon}{\boldsymbol{\upsilon}}\newcommand{\biphi}{\boldsymbol{\phi}}\newcommand{\bichi}{\boldsymbol{\chi}}\newcommand{\bipsi}{\boldsymbol{\psi}}\newcommand{\biomega}{\boldsymbol{\omega}}F\left( {1.12,8.99} \right) = 6.64,{\rm{\ }}P = 0.03$). When the pedestrian first appeared within the residual central field, the detection rate was not different across the viewing conditions, whereas when the pedestrian was in the prism-expanded field, the PFL+PR condition showed significantly higher detection rates than the simulated PFL condition ($\newcommand{\bialpha}{\boldsymbol{\alpha}}\newcommand{\bibeta}{\boldsymbol{\beta}}\newcommand{\bigamma}{\boldsymbol{\gamma}}\newcommand{\bidelta}{\boldsymbol{\delta}}\newcommand{\bivarepsilon}{\boldsymbol{\varepsilon}}\newcommand{\bizeta}{\boldsymbol{\zeta}}\newcommand{\bieta}{\boldsymbol{\eta}}\newcommand{\bitheta}{\boldsymbol{\theta}}\newcommand{\biiota}{\boldsymbol{\iota}}\newcommand{\bikappa}{\boldsymbol{\kappa}}\newcommand{\bilambda}{\boldsymbol{\lambda}}\newcommand{\bimu}{\boldsymbol{\mu}}\newcommand{\binu}{\boldsymbol{\nu}}\newcommand{\bixi}{\boldsymbol{\xi}}\newcommand{\biomicron}{\boldsymbol{\micron}}\newcommand{\bipi}{\boldsymbol{\pi}}\newcommand{\birho}{\boldsymbol{\rho}}\newcommand{\bisigma}{\boldsymbol{\sigma}}\newcommand{\bitau}{\boldsymbol{\tau}}\newcommand{\biupsilon}{\boldsymbol{\upsilon}}\newcommand{\biphi}{\boldsymbol{\phi}}\newcommand{\bichi}{\boldsymbol{\chi}}\newcommand{\bipsi}{\boldsymbol{\psi}}\newcommand{\biomega}{\boldsymbol{\omega}}t\left( {32} \right) = 3.9,{\rm{\ }}P = 0.001$), and the PFL+PR condition was not different from the NV condition ($\newcommand{\bialpha}{\boldsymbol{\alpha}}\newcommand{\bibeta}{\boldsymbol{\beta}}\newcommand{\bigamma}{\boldsymbol{\gamma}}\newcommand{\bidelta}{\boldsymbol{\delta}}\newcommand{\bivarepsilon}{\boldsymbol{\varepsilon}}\newcommand{\bizeta}{\boldsymbol{\zeta}}\newcommand{\bieta}{\boldsymbol{\eta}}\newcommand{\bitheta}{\boldsymbol{\theta}}\newcommand{\biiota}{\boldsymbol{\iota}}\newcommand{\bikappa}{\boldsymbol{\kappa}}\newcommand{\bilambda}{\boldsymbol{\lambda}}\newcommand{\bimu}{\boldsymbol{\mu}}\newcommand{\binu}{\boldsymbol{\nu}}\newcommand{\bixi}{\boldsymbol{\xi}}\newcommand{\biomicron}{\boldsymbol{\micron}}\newcommand{\bipi}{\boldsymbol{\pi}}\newcommand{\birho}{\boldsymbol{\rho}}\newcommand{\bisigma}{\boldsymbol{\sigma}}\newcommand{\bitau}{\boldsymbol{\tau}}\newcommand{\biupsilon}{\boldsymbol{\upsilon}}\newcommand{\biphi}{\boldsymbol{\phi}}\newcommand{\bichi}{\boldsymbol{\chi}}\newcommand{\bipsi}{\boldsymbol{\psi}}\newcommand{\biomega}{\boldsymbol{\omega}}t\left( {32} \right) = - 1.03,{\rm{\ }}P = 0.31$) (Fig. 6a). The collision decision accuracy was calculated among the detected pedestrians, which was slightly lower outside the residual central field in all viewing conditions, but the main effect of field of view positions was not significant ($\newcommand{\bialpha}{\boldsymbol{\alpha}}\newcommand{\bibeta}{\boldsymbol{\beta}}\newcommand{\bigamma}{\boldsymbol{\gamma}}\newcommand{\bidelta}{\boldsymbol{\delta}}\newcommand{\bivarepsilon}{\boldsymbol{\varepsilon}}\newcommand{\bizeta}{\boldsymbol{\zeta}}\newcommand{\bieta}{\boldsymbol{\eta}}\newcommand{\bitheta}{\boldsymbol{\theta}}\newcommand{\biiota}{\boldsymbol{\iota}}\newcommand{\bikappa}{\boldsymbol{\kappa}}\newcommand{\bilambda}{\boldsymbol{\lambda}}\newcommand{\bimu}{\boldsymbol{\mu}}\newcommand{\binu}{\boldsymbol{\nu}}\newcommand{\bixi}{\boldsymbol{\xi}}\newcommand{\biomicron}{\boldsymbol{\micron}}\newcommand{\bipi}{\boldsymbol{\pi}}\newcommand{\birho}{\boldsymbol{\rho}}\newcommand{\bisigma}{\boldsymbol{\sigma}}\newcommand{\bitau}{\boldsymbol{\tau}}\newcommand{\biupsilon}{\boldsymbol{\upsilon}}\newcommand{\biphi}{\boldsymbol{\phi}}\newcommand{\bichi}{\boldsymbol{\chi}}\newcommand{\bipsi}{\boldsymbol{\psi}}\newcommand{\biomega}{\boldsymbol{\omega}}F\left( {1,8} \right) = 0.51,\ P = 0.5$), and no significant posthoc contrast of any kind was found (Fig. 6b).

As for the detection RT (Fig. 6c), there was an interaction between field of view position and viewing condition: $\newcommand{\bialpha}{\boldsymbol{\alpha}}\newcommand{\bibeta}{\boldsymbol{\beta}}\newcommand{\bigamma}{\boldsymbol{\gamma}}\newcommand{\bidelta}{\boldsymbol{\delta}}\newcommand{\bivarepsilon}{\boldsymbol{\varepsilon}}\newcommand{\bizeta}{\boldsymbol{\zeta}}\newcommand{\bieta}{\boldsymbol{\eta}}\newcommand{\bitheta}{\boldsymbol{\theta}}\newcommand{\biiota}{\boldsymbol{\iota}}\newcommand{\bikappa}{\boldsymbol{\kappa}}\newcommand{\bilambda}{\boldsymbol{\lambda}}\newcommand{\bimu}{\boldsymbol{\mu}}\newcommand{\binu}{\boldsymbol{\nu}}\newcommand{\bixi}{\boldsymbol{\xi}}\newcommand{\biomicron}{\boldsymbol{\micron}}\newcommand{\bipi}{\boldsymbol{\pi}}\newcommand{\birho}{\boldsymbol{\rho}}\newcommand{\bisigma}{\boldsymbol{\sigma}}\newcommand{\bitau}{\boldsymbol{\tau}}\newcommand{\biupsilon}{\boldsymbol{\upsilon}}\newcommand{\biphi}{\boldsymbol{\phi}}\newcommand{\bichi}{\boldsymbol{\chi}}\newcommand{\bipsi}{\boldsymbol{\psi}}\newcommand{\biomega}{\boldsymbol{\omega}}F\left( {1.19,9.50} \right) = 11.45,\ P = 0.006$. The posthoc contrast showed that when the initial pedestrian position was within the residual central field, the detection RT was not different across the viewing conditions; when the initial bearing was larger than 10° (in the prism-expanded field), the detection RT for PFL+PR was shorter than that for PFL by about 1.2 seconds ($\newcommand{\bialpha}{\boldsymbol{\alpha}}\newcommand{\bibeta}{\boldsymbol{\beta}}\newcommand{\bigamma}{\boldsymbol{\gamma}}\newcommand{\bidelta}{\boldsymbol{\delta}}\newcommand{\bivarepsilon}{\boldsymbol{\varepsilon}}\newcommand{\bizeta}{\boldsymbol{\zeta}}\newcommand{\bieta}{\boldsymbol{\eta}}\newcommand{\bitheta}{\boldsymbol{\theta}}\newcommand{\biiota}{\boldsymbol{\iota}}\newcommand{\bikappa}{\boldsymbol{\kappa}}\newcommand{\bilambda}{\boldsymbol{\lambda}}\newcommand{\bimu}{\boldsymbol{\mu}}\newcommand{\binu}{\boldsymbol{\nu}}\newcommand{\bixi}{\boldsymbol{\xi}}\newcommand{\biomicron}{\boldsymbol{\micron}}\newcommand{\bipi}{\boldsymbol{\pi}}\newcommand{\birho}{\boldsymbol{\rho}}\newcommand{\bisigma}{\boldsymbol{\sigma}}\newcommand{\bitau}{\boldsymbol{\tau}}\newcommand{\biupsilon}{\boldsymbol{\upsilon}}\newcommand{\biphi}{\boldsymbol{\phi}}\newcommand{\bichi}{\boldsymbol{\chi}}\newcommand{\bipsi}{\boldsymbol{\psi}}\newcommand{\biomega}{\boldsymbol{\omega}}t\left( {32} \right) = - 3.57,\ P = 0.006$), though longer than the detection RT for NV by about 1.2 seconds ($\newcommand{\bialpha}{\boldsymbol{\alpha}}\newcommand{\bibeta}{\boldsymbol{\beta}}\newcommand{\bigamma}{\boldsymbol{\gamma}}\newcommand{\bidelta}{\boldsymbol{\delta}}\newcommand{\bivarepsilon}{\boldsymbol{\varepsilon}}\newcommand{\bizeta}{\boldsymbol{\zeta}}\newcommand{\bieta}{\boldsymbol{\eta}}\newcommand{\bitheta}{\boldsymbol{\theta}}\newcommand{\biiota}{\boldsymbol{\iota}}\newcommand{\bikappa}{\boldsymbol{\kappa}}\newcommand{\bilambda}{\boldsymbol{\lambda}}\newcommand{\bimu}{\boldsymbol{\mu}}\newcommand{\binu}{\boldsymbol{\nu}}\newcommand{\bixi}{\boldsymbol{\xi}}\newcommand{\biomicron}{\boldsymbol{\micron}}\newcommand{\bipi}{\boldsymbol{\pi}}\newcommand{\birho}{\boldsymbol{\rho}}\newcommand{\bisigma}{\boldsymbol{\sigma}}\newcommand{\bitau}{\boldsymbol{\tau}}\newcommand{\biupsilon}{\boldsymbol{\upsilon}}\newcommand{\biphi}{\boldsymbol{\phi}}\newcommand{\bichi}{\boldsymbol{\chi}}\newcommand{\bipsi}{\boldsymbol{\psi}}\newcommand{\biomega}{\boldsymbol{\omega}}t\left( {32} \right) = 3.49,\ P = 0.006$). The time subjects spent reaching a collision decision (after detection) was also affected by both the viewing condition and the field of view position ($\newcommand{\bialpha}{\boldsymbol{\alpha}}\newcommand{\bibeta}{\boldsymbol{\beta}}\newcommand{\bigamma}{\boldsymbol{\gamma}}\newcommand{\bidelta}{\boldsymbol{\delta}}\newcommand{\bivarepsilon}{\boldsymbol{\varepsilon}}\newcommand{\bizeta}{\boldsymbol{\zeta}}\newcommand{\bieta}{\boldsymbol{\eta}}\newcommand{\bitheta}{\boldsymbol{\theta}}\newcommand{\biiota}{\boldsymbol{\iota}}\newcommand{\bikappa}{\boldsymbol{\kappa}}\newcommand{\bilambda}{\boldsymbol{\lambda}}\newcommand{\bimu}{\boldsymbol{\mu}}\newcommand{\binu}{\boldsymbol{\nu}}\newcommand{\bixi}{\boldsymbol{\xi}}\newcommand{\biomicron}{\boldsymbol{\micron}}\newcommand{\bipi}{\boldsymbol{\pi}}\newcommand{\birho}{\boldsymbol{\rho}}\newcommand{\bisigma}{\boldsymbol{\sigma}}\newcommand{\bitau}{\boldsymbol{\tau}}\newcommand{\biupsilon}{\boldsymbol{\upsilon}}\newcommand{\biphi}{\boldsymbol{\phi}}\newcommand{\bichi}{\boldsymbol{\chi}}\newcommand{\bipsi}{\boldsymbol{\psi}}\newcommand{\biomega}{\boldsymbol{\omega}}F\left( {1.75,13.98} \right) = 6.81,\ P = 0.01$). Within the residual central field, no difference in decision RT was observed across the viewing conditions; in the prism-expanded field, the decision RT was the shortest for simulated PFL—about 0.6 seconds shorter than NV ($\newcommand{\bialpha}{\boldsymbol{\alpha}}\newcommand{\bibeta}{\boldsymbol{\beta}}\newcommand{\bigamma}{\boldsymbol{\gamma}}\newcommand{\bidelta}{\boldsymbol{\delta}}\newcommand{\bivarepsilon}{\boldsymbol{\varepsilon}}\newcommand{\bizeta}{\boldsymbol{\zeta}}\newcommand{\bieta}{\boldsymbol{\eta}}\newcommand{\bitheta}{\boldsymbol{\theta}}\newcommand{\biiota}{\boldsymbol{\iota}}\newcommand{\bikappa}{\boldsymbol{\kappa}}\newcommand{\bilambda}{\boldsymbol{\lambda}}\newcommand{\bimu}{\boldsymbol{\mu}}\newcommand{\binu}{\boldsymbol{\nu}}\newcommand{\bixi}{\boldsymbol{\xi}}\newcommand{\biomicron}{\boldsymbol{\micron}}\newcommand{\bipi}{\boldsymbol{\pi}}\newcommand{\birho}{\boldsymbol{\rho}}\newcommand{\bisigma}{\boldsymbol{\sigma}}\newcommand{\bitau}{\boldsymbol{\tau}}\newcommand{\biupsilon}{\boldsymbol{\upsilon}}\newcommand{\biphi}{\boldsymbol{\phi}}\newcommand{\bichi}{\boldsymbol{\chi}}\newcommand{\bipsi}{\boldsymbol{\psi}}\newcommand{\biomega}{\boldsymbol{\omega}}t\left( {28.01} \right) = 4.01,\ P = 0.0025$) and 0.4 seconds shorter than PFL+PR ($\newcommand{\bialpha}{\boldsymbol{\alpha}}\newcommand{\bibeta}{\boldsymbol{\beta}}\newcommand{\bigamma}{\boldsymbol{\gamma}}\newcommand{\bidelta}{\boldsymbol{\delta}}\newcommand{\bivarepsilon}{\boldsymbol{\varepsilon}}\newcommand{\bizeta}{\boldsymbol{\zeta}}\newcommand{\bieta}{\boldsymbol{\eta}}\newcommand{\bitheta}{\boldsymbol{\theta}}\newcommand{\biiota}{\boldsymbol{\iota}}\newcommand{\bikappa}{\boldsymbol{\kappa}}\newcommand{\bilambda}{\boldsymbol{\lambda}}\newcommand{\bimu}{\boldsymbol{\mu}}\newcommand{\binu}{\boldsymbol{\nu}}\newcommand{\bixi}{\boldsymbol{\xi}}\newcommand{\biomicron}{\boldsymbol{\micron}}\newcommand{\bipi}{\boldsymbol{\pi}}\newcommand{\birho}{\boldsymbol{\rho}}\newcommand{\bisigma}{\boldsymbol{\sigma}}\newcommand{\bitau}{\boldsymbol{\tau}}\newcommand{\biupsilon}{\boldsymbol{\upsilon}}\newcommand{\biphi}{\boldsymbol{\phi}}\newcommand{\bichi}{\boldsymbol{\chi}}\newcommand{\bipsi}{\boldsymbol{\psi}}\newcommand{\biomega}{\boldsymbol{\omega}}t\left( {28.01} \right) = 2.86,\ P = 0.04$), while no significant difference was found between PFL+PR and NV. The total RT (the detection RT plus the decision RT) showed a moderate interaction between viewing condition and field of view position ($\newcommand{\bialpha}{\boldsymbol{\alpha}}\newcommand{\bibeta}{\boldsymbol{\beta}}\newcommand{\bigamma}{\boldsymbol{\gamma}}\newcommand{\bidelta}{\boldsymbol{\delta}}\newcommand{\bivarepsilon}{\boldsymbol{\varepsilon}}\newcommand{\bizeta}{\boldsymbol{\zeta}}\newcommand{\bieta}{\boldsymbol{\eta}}\newcommand{\bitheta}{\boldsymbol{\theta}}\newcommand{\biiota}{\boldsymbol{\iota}}\newcommand{\bikappa}{\boldsymbol{\kappa}}\newcommand{\bilambda}{\boldsymbol{\lambda}}\newcommand{\bimu}{\boldsymbol{\mu}}\newcommand{\binu}{\boldsymbol{\nu}}\newcommand{\bixi}{\boldsymbol{\xi}}\newcommand{\biomicron}{\boldsymbol{\micron}}\newcommand{\bipi}{\boldsymbol{\pi}}\newcommand{\birho}{\boldsymbol{\rho}}\newcommand{\bisigma}{\boldsymbol{\sigma}}\newcommand{\bitau}{\boldsymbol{\tau}}\newcommand{\biupsilon}{\boldsymbol{\upsilon}}\newcommand{\biphi}{\boldsymbol{\phi}}\newcommand{\bichi}{\boldsymbol{\chi}}\newcommand{\bipsi}{\boldsymbol{\psi}}\newcommand{\biomega}{\boldsymbol{\omega}}F\left( {1.42,11.40} \right) = 7.69,\ P = 0.01$). Within the residual central field, the effect of viewing condition was minimal; however, in the prism-expanded field, although total RTs were about 1.0 second longer in the PFL+PR condition than the NV condition ($\newcommand{\bialpha}{\boldsymbol{\alpha}}\newcommand{\bibeta}{\boldsymbol{\beta}}\newcommand{\bigamma}{\boldsymbol{\gamma}}\newcommand{\bidelta}{\boldsymbol{\delta}}\newcommand{\bivarepsilon}{\boldsymbol{\varepsilon}}\newcommand{\bizeta}{\boldsymbol{\zeta}}\newcommand{\bieta}{\boldsymbol{\eta}}\newcommand{\bitheta}{\boldsymbol{\theta}}\newcommand{\biiota}{\boldsymbol{\iota}}\newcommand{\bikappa}{\boldsymbol{\kappa}}\newcommand{\bilambda}{\boldsymbol{\lambda}}\newcommand{\bimu}{\boldsymbol{\mu}}\newcommand{\binu}{\boldsymbol{\nu}}\newcommand{\bixi}{\boldsymbol{\xi}}\newcommand{\biomicron}{\boldsymbol{\micron}}\newcommand{\bipi}{\boldsymbol{\pi}}\newcommand{\birho}{\boldsymbol{\rho}}\newcommand{\bisigma}{\boldsymbol{\sigma}}\newcommand{\bitau}{\boldsymbol{\tau}}\newcommand{\biupsilon}{\boldsymbol{\upsilon}}\newcommand{\biphi}{\boldsymbol{\phi}}\newcommand{\bichi}{\boldsymbol{\chi}}\newcommand{\bipsi}{\boldsymbol{\psi}}\newcommand{\biomega}{\boldsymbol{\omega}}t\left( {31.2} \right) = 3.03,\ P = 0.025$), they were improved when compared with the simulated PFL condition, which were about 1.8 seconds longer than in the NV condition ($\newcommand{\bialpha}{\boldsymbol{\alpha}}\newcommand{\bibeta}{\boldsymbol{\beta}}\newcommand{\bigamma}{\boldsymbol{\gamma}}\newcommand{\bidelta}{\boldsymbol{\delta}}\newcommand{\bivarepsilon}{\boldsymbol{\varepsilon}}\newcommand{\bizeta}{\boldsymbol{\zeta}}\newcommand{\bieta}{\boldsymbol{\eta}}\newcommand{\bitheta}{\boldsymbol{\theta}}\newcommand{\biiota}{\boldsymbol{\iota}}\newcommand{\bikappa}{\boldsymbol{\kappa}}\newcommand{\bilambda}{\boldsymbol{\lambda}}\newcommand{\bimu}{\boldsymbol{\mu}}\newcommand{\binu}{\boldsymbol{\nu}}\newcommand{\bixi}{\boldsymbol{\xi}}\newcommand{\biomicron}{\boldsymbol{\micron}}\newcommand{\bipi}{\boldsymbol{\pi}}\newcommand{\birho}{\boldsymbol{\rho}}\newcommand{\bisigma}{\boldsymbol{\sigma}}\newcommand{\bitau}{\boldsymbol{\tau}}\newcommand{\biupsilon}{\boldsymbol{\upsilon}}\newcommand{\biphi}{\boldsymbol{\phi}}\newcommand{\bichi}{\boldsymbol{\chi}}\newcommand{\bipsi}{\boldsymbol{\psi}}\newcommand{\biomega}{\boldsymbol{\omega}}t\left( {31.2} \right) = 5.39,\ P \lt 0.0001$).

For the eight patients with PFL, when the pedestrians initially appeared within their residual central fields, the average detection rate was 95% (Fig. 6a, marked as PAT). For the conditions with $\newcommand{\bialpha}{\boldsymbol{\alpha}}\newcommand{\bibeta}{\boldsymbol{\beta}}\newcommand{\bigamma}{\boldsymbol{\gamma}}\newcommand{\bidelta}{\boldsymbol{\delta}}\newcommand{\bivarepsilon}{\boldsymbol{\varepsilon}}\newcommand{\bizeta}{\boldsymbol{\zeta}}\newcommand{\bieta}{\boldsymbol{\eta}}\newcommand{\bitheta}{\boldsymbol{\theta}}\newcommand{\biiota}{\boldsymbol{\iota}}\newcommand{\bikappa}{\boldsymbol{\kappa}}\newcommand{\bilambda}{\boldsymbol{\lambda}}\newcommand{\bimu}{\boldsymbol{\mu}}\newcommand{\binu}{\boldsymbol{\nu}}\newcommand{\bixi}{\boldsymbol{\xi}}\newcommand{\biomicron}{\boldsymbol{\micron}}\newcommand{\bipi}{\boldsymbol{\pi}}\newcommand{\birho}{\boldsymbol{\rho}}\newcommand{\bisigma}{\boldsymbol{\sigma}}\newcommand{\bitau}{\boldsymbol{\tau}}\newcommand{\biupsilon}{\boldsymbol{\upsilon}}\newcommand{\biphi}{\boldsymbol{\phi}}\newcommand{\bichi}{\boldsymbol{\chi}}\newcommand{\bipsi}{\boldsymbol{\psi}}\newcommand{\biomega}{\boldsymbol{\omega}}{\beta _{{\rm{t}} = 0}} = 30^{\circ}$, the pedestrian detection rate dropped significantly to 59% on average (Bonferroni corrected *P* < 0.0001). The one-way within-subject ANOVA showed a significant effect of field of view positions on detection rates (*F*(1, 78) = 31.9, *P* < 0.0001). The detection RTs were also significantly different (*F*(1, 70) = 42.5, *P* < 0.0001): the average detection RT of the conditions within the central field was about 2.6 seconds, but the detection RT of the conditions with a 30° initial bearing was 4.8 seconds. To compare the patients' results with the simulated PFL results in normally sighted subjects, two-way between-subjects ANOVAs (participants type × field of view positions) were conducted. Neither the detection rates (*F*(1, 30) = 1.55, *P* = 0.22) nor the collision decision accuracy (*F*(1, 30) = 2.11, *P* = 0.16) across the field of view positions were significantly different between the two participant groups. However, the patients had longer detection RTs (*F*(1, 30) = 6.24, *P* = 0.018), and subsequently, shorter decision RTs (*F*(1, 30) = 5.83, *P* = 0.022) than the subjects with simulated PFL. The detection rate and RT for the patients with real PFL and their collision decision accuracy were largely similar to those observed among the normally sighted subjects with simulated PFL, although the performance of the patients with real PFL was slightly worse: longer detection RTs when $\newcommand{\bialpha}{\boldsymbol{\alpha}}\newcommand{\bibeta}{\boldsymbol{\beta}}\newcommand{\bigamma}{\boldsymbol{\gamma}}\newcommand{\bidelta}{\boldsymbol{\delta}}\newcommand{\bivarepsilon}{\boldsymbol{\varepsilon}}\newcommand{\bizeta}{\boldsymbol{\zeta}}\newcommand{\bieta}{\boldsymbol{\eta}}\newcommand{\bitheta}{\boldsymbol{\theta}}\newcommand{\biiota}{\boldsymbol{\iota}}\newcommand{\bikappa}{\boldsymbol{\kappa}}\newcommand{\bilambda}{\boldsymbol{\lambda}}\newcommand{\bimu}{\boldsymbol{\mu}}\newcommand{\binu}{\boldsymbol{\nu}}\newcommand{\bixi}{\boldsymbol{\xi}}\newcommand{\biomicron}{\boldsymbol{\micron}}\newcommand{\bipi}{\boldsymbol{\pi}}\newcommand{\birho}{\boldsymbol{\rho}}\newcommand{\bisigma}{\boldsymbol{\sigma}}\newcommand{\bitau}{\boldsymbol{\tau}}\newcommand{\biupsilon}{\boldsymbol{\upsilon}}\newcommand{\biphi}{\boldsymbol{\phi}}\newcommand{\bichi}{\boldsymbol{\chi}}\newcommand{\bipsi}{\boldsymbol{\psi}}\newcommand{\biomega}{\boldsymbol{\omega}}{\beta _{{\rm{t}} = 0}} \le 10^{\circ}$ or $\newcommand{\bialpha}{\boldsymbol{\alpha}}\newcommand{\bibeta}{\boldsymbol{\beta}}\newcommand{\bigamma}{\boldsymbol{\gamma}}\newcommand{\bidelta}{\boldsymbol{\delta}}\newcommand{\bivarepsilon}{\boldsymbol{\varepsilon}}\newcommand{\bizeta}{\boldsymbol{\zeta}}\newcommand{\bieta}{\boldsymbol{\eta}}\newcommand{\bitheta}{\boldsymbol{\theta}}\newcommand{\biiota}{\boldsymbol{\iota}}\newcommand{\bikappa}{\boldsymbol{\kappa}}\newcommand{\bilambda}{\boldsymbol{\lambda}}\newcommand{\bimu}{\boldsymbol{\mu}}\newcommand{\binu}{\boldsymbol{\nu}}\newcommand{\bixi}{\boldsymbol{\xi}}\newcommand{\biomicron}{\boldsymbol{\micron}}\newcommand{\bipi}{\boldsymbol{\pi}}\newcommand{\birho}{\boldsymbol{\rho}}\newcommand{\bisigma}{\boldsymbol{\sigma}}\newcommand{\bitau}{\boldsymbol{\tau}}\newcommand{\biupsilon}{\boldsymbol{\upsilon}}\newcommand{\biphi}{\boldsymbol{\phi}}\newcommand{\bichi}{\boldsymbol{\chi}}\newcommand{\bipsi}{\boldsymbol{\psi}}\newcommand{\biomega}{\boldsymbol{\omega}}{\beta _{{\rm{t}} = 0}} = 30^{\circ}$ (Fig. 6).

### Detection Rate, Collision Decision, and RT for Each Condition

**Figure 7:**
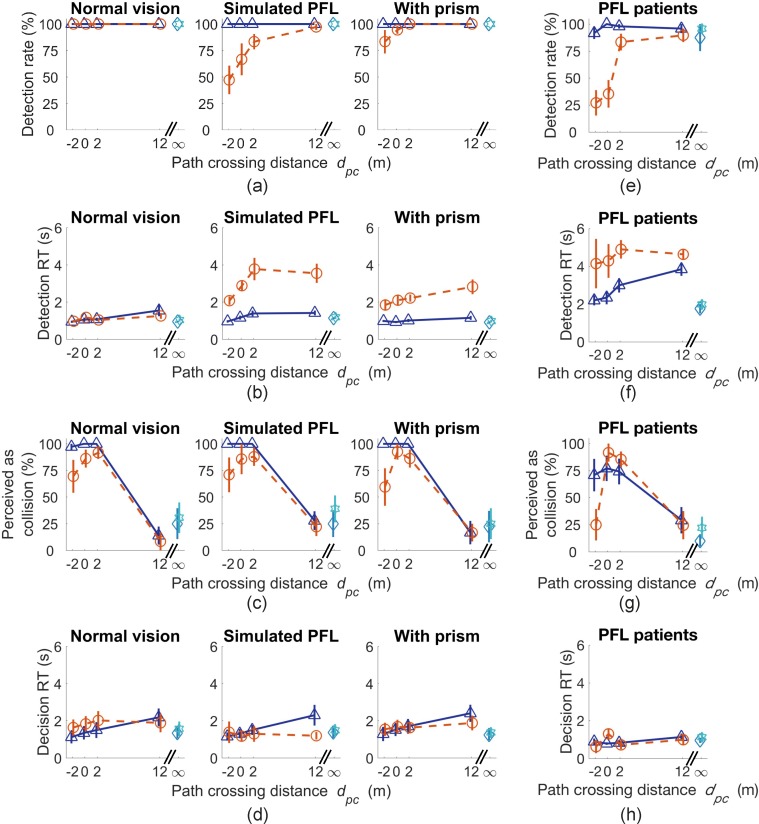
The average detection rate, detection RT, decision of collision, and decision RT results from the normally sighted subjects (a–d) and the PFL patients (e–h). The three panels in (a–d) are for the three viewing conditions as marked. The blue symbols indicate the conditions when pedestrians initially appear with $\newcommand{\bialpha}{\boldsymbol{\alpha}}\newcommand{\bibeta}{\boldsymbol{\beta}}\newcommand{\bigamma}{\boldsymbol{\gamma}}\newcommand{\bidelta}{\boldsymbol{\delta}}\newcommand{\bivarepsilon}{\boldsymbol{\varepsilon}}\newcommand{\bizeta}{\boldsymbol{\zeta}}\newcommand{\bieta}{\boldsymbol{\eta}}\newcommand{\bitheta}{\boldsymbol{\theta}}\newcommand{\biiota}{\boldsymbol{\iota}}\newcommand{\bikappa}{\boldsymbol{\kappa}}\newcommand{\bilambda}{\boldsymbol{\lambda}}\newcommand{\bimu}{\boldsymbol{\mu}}\newcommand{\binu}{\boldsymbol{\nu}}\newcommand{\bixi}{\boldsymbol{\xi}}\newcommand{\biomicron}{\boldsymbol{\micron}}\newcommand{\bipi}{\boldsymbol{\pi}}\newcommand{\birho}{\boldsymbol{\rho}}\newcommand{\bisigma}{\boldsymbol{\sigma}}\newcommand{\bitau}{\boldsymbol{\tau}}\newcommand{\biupsilon}{\boldsymbol{\upsilon}}\newcommand{\biphi}{\boldsymbol{\phi}}\newcommand{\bichi}{\boldsymbol{\chi}}\newcommand{\bipsi}{\boldsymbol{\psi}}\newcommand{\biomega}{\boldsymbol{\omega}}{\beta _{t = 0}} \le 10^{\circ}$ (triangles, $\newcommand{\bialpha}{\boldsymbol{\alpha}}\newcommand{\bibeta}{\boldsymbol{\beta}}\newcommand{\bigamma}{\boldsymbol{\gamma}}\newcommand{\bidelta}{\boldsymbol{\delta}}\newcommand{\bivarepsilon}{\boldsymbol{\varepsilon}}\newcommand{\bizeta}{\boldsymbol{\zeta}}\newcommand{\bieta}{\boldsymbol{\eta}}\newcommand{\bitheta}{\boldsymbol{\theta}}\newcommand{\biiota}{\boldsymbol{\iota}}\newcommand{\bikappa}{\boldsymbol{\kappa}}\newcommand{\bilambda}{\boldsymbol{\lambda}}\newcommand{\bimu}{\boldsymbol{\mu}}\newcommand{\binu}{\boldsymbol{\nu}}\newcommand{\bixi}{\boldsymbol{\xi}}\newcommand{\biomicron}{\boldsymbol{\micron}}\newcommand{\bipi}{\boldsymbol{\pi}}\newcommand{\birho}{\boldsymbol{\rho}}\newcommand{\bisigma}{\boldsymbol{\sigma}}\newcommand{\bitau}{\boldsymbol{\tau}}\newcommand{\biupsilon}{\boldsymbol{\upsilon}}\newcommand{\biphi}{\boldsymbol{\phi}}\newcommand{\bichi}{\boldsymbol{\chi}}\newcommand{\bipsi}{\boldsymbol{\psi}}\newcommand{\biomega}{\boldsymbol{\omega}}{\beta _{t = 0}} = 10^{\circ}$; diamond, $\newcommand{\bialpha}{\boldsymbol{\alpha}}\newcommand{\bibeta}{\boldsymbol{\beta}}\newcommand{\bigamma}{\boldsymbol{\gamma}}\newcommand{\bidelta}{\boldsymbol{\delta}}\newcommand{\bivarepsilon}{\boldsymbol{\varepsilon}}\newcommand{\bizeta}{\boldsymbol{\zeta}}\newcommand{\bieta}{\boldsymbol{\eta}}\newcommand{\bitheta}{\boldsymbol{\theta}}\newcommand{\biiota}{\boldsymbol{\iota}}\newcommand{\bikappa}{\boldsymbol{\kappa}}\newcommand{\bilambda}{\boldsymbol{\lambda}}\newcommand{\bimu}{\boldsymbol{\mu}}\newcommand{\binu}{\boldsymbol{\nu}}\newcommand{\bixi}{\boldsymbol{\xi}}\newcommand{\biomicron}{\boldsymbol{\micron}}\newcommand{\bipi}{\boldsymbol{\pi}}\newcommand{\birho}{\boldsymbol{\rho}}\newcommand{\bisigma}{\boldsymbol{\sigma}}\newcommand{\bitau}{\boldsymbol{\tau}}\newcommand{\biupsilon}{\boldsymbol{\upsilon}}\newcommand{\biphi}{\boldsymbol{\phi}}\newcommand{\bichi}{\boldsymbol{\chi}}\newcommand{\bipsi}{\boldsymbol{\psi}}\newcommand{\biomega}{\boldsymbol{\omega}}{\beta _{t = 0}} = 5^{\circ}$; star, $\newcommand{\bialpha}{\boldsymbol{\alpha}}\newcommand{\bibeta}{\boldsymbol{\beta}}\newcommand{\bigamma}{\boldsymbol{\gamma}}\newcommand{\bidelta}{\boldsymbol{\delta}}\newcommand{\bivarepsilon}{\boldsymbol{\varepsilon}}\newcommand{\bizeta}{\boldsymbol{\zeta}}\newcommand{\bieta}{\boldsymbol{\eta}}\newcommand{\bitheta}{\boldsymbol{\theta}}\newcommand{\biiota}{\boldsymbol{\iota}}\newcommand{\bikappa}{\boldsymbol{\kappa}}\newcommand{\bilambda}{\boldsymbol{\lambda}}\newcommand{\bimu}{\boldsymbol{\mu}}\newcommand{\binu}{\boldsymbol{\nu}}\newcommand{\bixi}{\boldsymbol{\xi}}\newcommand{\biomicron}{\boldsymbol{\micron}}\newcommand{\bipi}{\boldsymbol{\pi}}\newcommand{\birho}{\boldsymbol{\rho}}\newcommand{\bisigma}{\boldsymbol{\sigma}}\newcommand{\bitau}{\boldsymbol{\tau}}\newcommand{\biupsilon}{\boldsymbol{\upsilon}}\newcommand{\biphi}{\boldsymbol{\phi}}\newcommand{\bichi}{\boldsymbol{\chi}}\newcommand{\bipsi}{\boldsymbol{\psi}}\newcommand{\biomega}{\boldsymbol{\omega}}{\beta _{t = 0}} = 2.5^{\circ}$), the orange circles are for the conditions with $\newcommand{\bialpha}{\boldsymbol{\alpha}}\newcommand{\bibeta}{\boldsymbol{\beta}}\newcommand{\bigamma}{\boldsymbol{\gamma}}\newcommand{\bidelta}{\boldsymbol{\delta}}\newcommand{\bivarepsilon}{\boldsymbol{\varepsilon}}\newcommand{\bizeta}{\boldsymbol{\zeta}}\newcommand{\bieta}{\boldsymbol{\eta}}\newcommand{\bitheta}{\boldsymbol{\theta}}\newcommand{\biiota}{\boldsymbol{\iota}}\newcommand{\bikappa}{\boldsymbol{\kappa}}\newcommand{\bilambda}{\boldsymbol{\lambda}}\newcommand{\bimu}{\boldsymbol{\mu}}\newcommand{\binu}{\boldsymbol{\nu}}\newcommand{\bixi}{\boldsymbol{\xi}}\newcommand{\biomicron}{\boldsymbol{\micron}}\newcommand{\bipi}{\boldsymbol{\pi}}\newcommand{\birho}{\boldsymbol{\rho}}\newcommand{\bisigma}{\boldsymbol{\sigma}}\newcommand{\bitau}{\boldsymbol{\tau}}\newcommand{\biupsilon}{\boldsymbol{\upsilon}}\newcommand{\biphi}{\boldsymbol{\phi}}\newcommand{\bichi}{\boldsymbol{\chi}}\newcommand{\bipsi}{\boldsymbol{\psi}}\newcommand{\biomega}{\boldsymbol{\omega}}{\beta _{t = 0}} = 30^{\circ}$. The x-axis shows the path crossing distance, $\newcommand{\bialpha}{\boldsymbol{\alpha}}\newcommand{\bibeta}{\boldsymbol{\beta}}\newcommand{\bigamma}{\boldsymbol{\gamma}}\newcommand{\bidelta}{\boldsymbol{\delta}}\newcommand{\bivarepsilon}{\boldsymbol{\varepsilon}}\newcommand{\bizeta}{\boldsymbol{\zeta}}\newcommand{\bieta}{\boldsymbol{\eta}}\newcommand{\bitheta}{\boldsymbol{\theta}}\newcommand{\biiota}{\boldsymbol{\iota}}\newcommand{\bikappa}{\boldsymbol{\kappa}}\newcommand{\bilambda}{\boldsymbol{\lambda}}\newcommand{\bimu}{\boldsymbol{\mu}}\newcommand{\binu}{\boldsymbol{\nu}}\newcommand{\bixi}{\boldsymbol{\xi}}\newcommand{\biomicron}{\boldsymbol{\micron}}\newcommand{\bipi}{\boldsymbol{\pi}}\newcommand{\birho}{\boldsymbol{\rho}}\newcommand{\bisigma}{\boldsymbol{\sigma}}\newcommand{\bitau}{\boldsymbol{\tau}}\newcommand{\biupsilon}{\boldsymbol{\upsilon}}\newcommand{\biphi}{\boldsymbol{\phi}}\newcommand{\bichi}{\boldsymbol{\chi}}\newcommand{\bipsi}{\boldsymbol{\psi}}\newcommand{\biomega}{\boldsymbol{\omega}}{d_{pc}}$ = −2 m, 0, +2 m, +12 m, and symbol ∞ for $\newcommand{\bialpha}{\boldsymbol{\alpha}}\newcommand{\bibeta}{\boldsymbol{\beta}}\newcommand{\bigamma}{\boldsymbol{\gamma}}\newcommand{\bidelta}{\boldsymbol{\delta}}\newcommand{\bivarepsilon}{\boldsymbol{\varepsilon}}\newcommand{\bizeta}{\boldsymbol{\zeta}}\newcommand{\bieta}{\boldsymbol{\eta}}\newcommand{\bitheta}{\boldsymbol{\theta}}\newcommand{\biiota}{\boldsymbol{\iota}}\newcommand{\bikappa}{\boldsymbol{\kappa}}\newcommand{\bilambda}{\boldsymbol{\lambda}}\newcommand{\bimu}{\boldsymbol{\mu}}\newcommand{\binu}{\boldsymbol{\nu}}\newcommand{\bixi}{\boldsymbol{\xi}}\newcommand{\biomicron}{\boldsymbol{\micron}}\newcommand{\bipi}{\boldsymbol{\pi}}\newcommand{\birho}{\boldsymbol{\rho}}\newcommand{\bisigma}{\boldsymbol{\sigma}}\newcommand{\bitau}{\boldsymbol{\tau}}\newcommand{\biupsilon}{\boldsymbol{\upsilon}}\newcommand{\biphi}{\boldsymbol{\phi}}\newcommand{\bichi}{\boldsymbol{\chi}}\newcommand{\bipsi}{\boldsymbol{\psi}}\newcommand{\biomega}{\boldsymbol{\omega}}{d_{pc}}$ indicates the sidewalk-like encounters. (a) The pedestrian detection rate of the normally sighted subjects for the individual conditions. (b) The RT to detect the pedestrians. (c) The percentage of trials perceived as collisions. (d) The RT to make the collision judgment (after the pedestrian detection). Error bars represent standard error of the mean. (e–h) The average detection rate, detection RT, decision of collision, and decision RT of the eight PFL patients. Their behavioral patterns are similar to those acquired in simulated PFL with the normally sighted subjects (middle column in a–d).

The performance for each condition was further evaluated. For the normally sighted subjects, among the pedestrians initially appearing at 30° eccentricity, those crossing behind the subject yielded the lowest detection rates; pedestrians passing in front were more likely to be detected (Fig. 7a). A three-way ANOVA (viewing condition × initial bearing × path crossing distance) showed a significant interaction between the viewing condition and the initial pedestrian bearing in the detection rate ($\newcommand{\bialpha}{\boldsymbol{\alpha}}\newcommand{\bibeta}{\boldsymbol{\beta}}\newcommand{\bigamma}{\boldsymbol{\gamma}}\newcommand{\bidelta}{\boldsymbol{\delta}}\newcommand{\bivarepsilon}{\boldsymbol{\varepsilon}}\newcommand{\bizeta}{\boldsymbol{\zeta}}\newcommand{\bieta}{\boldsymbol{\eta}}\newcommand{\bitheta}{\boldsymbol{\theta}}\newcommand{\biiota}{\boldsymbol{\iota}}\newcommand{\bikappa}{\boldsymbol{\kappa}}\newcommand{\bilambda}{\boldsymbol{\lambda}}\newcommand{\bimu}{\boldsymbol{\mu}}\newcommand{\binu}{\boldsymbol{\nu}}\newcommand{\bixi}{\boldsymbol{\xi}}\newcommand{\biomicron}{\boldsymbol{\micron}}\newcommand{\bipi}{\boldsymbol{\pi}}\newcommand{\birho}{\boldsymbol{\rho}}\newcommand{\bisigma}{\boldsymbol{\sigma}}\newcommand{\bitau}{\boldsymbol{\tau}}\newcommand{\biupsilon}{\boldsymbol{\upsilon}}\newcommand{\biphi}{\boldsymbol{\phi}}\newcommand{\bichi}{\boldsymbol{\chi}}\newcommand{\bipsi}{\boldsymbol{\psi}}\newcommand{\biomega}{\boldsymbol{\omega}}F\left( {2,142} \right) = 17.7,\ P \lt 0.0001$). The detection RT (Fig. 7b) was similar across all pedestrian conditions while viewing with NV (1.1 seconds). Across the viewing conditions, if the pedestrians initially appeared within the residual central field ($\newcommand{\bialpha}{\boldsymbol{\alpha}}\newcommand{\bibeta}{\boldsymbol{\beta}}\newcommand{\bigamma}{\boldsymbol{\gamma}}\newcommand{\bidelta}{\boldsymbol{\delta}}\newcommand{\bivarepsilon}{\boldsymbol{\varepsilon}}\newcommand{\bizeta}{\boldsymbol{\zeta}}\newcommand{\bieta}{\boldsymbol{\eta}}\newcommand{\bitheta}{\boldsymbol{\theta}}\newcommand{\biiota}{\boldsymbol{\iota}}\newcommand{\bikappa}{\boldsymbol{\kappa}}\newcommand{\bilambda}{\boldsymbol{\lambda}}\newcommand{\bimu}{\boldsymbol{\mu}}\newcommand{\binu}{\boldsymbol{\nu}}\newcommand{\bixi}{\boldsymbol{\xi}}\newcommand{\biomicron}{\boldsymbol{\micron}}\newcommand{\bipi}{\boldsymbol{\pi}}\newcommand{\birho}{\boldsymbol{\rho}}\newcommand{\bisigma}{\boldsymbol{\sigma}}\newcommand{\bitau}{\boldsymbol{\tau}}\newcommand{\biupsilon}{\boldsymbol{\upsilon}}\newcommand{\biphi}{\boldsymbol{\phi}}\newcommand{\bichi}{\boldsymbol{\chi}}\newcommand{\bipsi}{\boldsymbol{\psi}}\newcommand{\biomega}{\boldsymbol{\omega}}{\beta _{t = 0}} \le 10^{\circ}$), the detection RT was not different. Thus, no negative effects (on detection rate or RT) were observed within the residual central field when the prisms were placed. With simulated PFL, the subjects took much longer to detect pedestrians appearing at 30° bearing. The detection RT improved (reduced) with the prisms. The ANOVA showed a significant interaction between viewing condition and initial pedestrian bearing for detection RT ($\newcommand{\bialpha}{\boldsymbol{\alpha}}\newcommand{\bibeta}{\boldsymbol{\beta}}\newcommand{\bigamma}{\boldsymbol{\gamma}}\newcommand{\bidelta}{\boldsymbol{\delta}}\newcommand{\bivarepsilon}{\boldsymbol{\varepsilon}}\newcommand{\bizeta}{\boldsymbol{\zeta}}\newcommand{\bieta}{\boldsymbol{\eta}}\newcommand{\bitheta}{\boldsymbol{\theta}}\newcommand{\biiota}{\boldsymbol{\iota}}\newcommand{\bikappa}{\boldsymbol{\kappa}}\newcommand{\bilambda}{\boldsymbol{\lambda}}\newcommand{\bimu}{\boldsymbol{\mu}}\newcommand{\binu}{\boldsymbol{\nu}}\newcommand{\bixi}{\boldsymbol{\xi}}\newcommand{\biomicron}{\boldsymbol{\micron}}\newcommand{\bipi}{\boldsymbol{\pi}}\newcommand{\birho}{\boldsymbol{\rho}}\newcommand{\bisigma}{\boldsymbol{\sigma}}\newcommand{\bitau}{\boldsymbol{\tau}}\newcommand{\biupsilon}{\boldsymbol{\upsilon}}\newcommand{\biphi}{\boldsymbol{\phi}}\newcommand{\bichi}{\boldsymbol{\chi}}\newcommand{\bipsi}{\boldsymbol{\psi}}\newcommand{\biomega}{\boldsymbol{\omega}}F\left( {2,136} \right) = 47.5,\ P \lt 0.0001$). A detection RT was only available when the pedestrian was in fact detected; therefore, Figures 7a and 7b are meant to be interpreted together. A relatively high detection rate but a very long detection RT does not indicate overall good detection performance.

As for subjects' pedestrian collision decisions, the conditions with $\newcommand{\bialpha}{\boldsymbol{\alpha}}\newcommand{\bibeta}{\boldsymbol{\beta}}\newcommand{\bigamma}{\boldsymbol{\gamma}}\newcommand{\bidelta}{\boldsymbol{\delta}}\newcommand{\bivarepsilon}{\boldsymbol{\varepsilon}}\newcommand{\bizeta}{\boldsymbol{\zeta}}\newcommand{\bieta}{\boldsymbol{\eta}}\newcommand{\bitheta}{\boldsymbol{\theta}}\newcommand{\biiota}{\boldsymbol{\iota}}\newcommand{\bikappa}{\boldsymbol{\kappa}}\newcommand{\bilambda}{\boldsymbol{\lambda}}\newcommand{\bimu}{\boldsymbol{\mu}}\newcommand{\binu}{\boldsymbol{\nu}}\newcommand{\bixi}{\boldsymbol{\xi}}\newcommand{\biomicron}{\boldsymbol{\micron}}\newcommand{\bipi}{\boldsymbol{\pi}}\newcommand{\birho}{\boldsymbol{\rho}}\newcommand{\bisigma}{\boldsymbol{\sigma}}\newcommand{\bitau}{\boldsymbol{\tau}}\newcommand{\biupsilon}{\boldsymbol{\upsilon}}\newcommand{\biphi}{\boldsymbol{\phi}}\newcommand{\bichi}{\boldsymbol{\chi}}\newcommand{\bipsi}{\boldsymbol{\psi}}\newcommand{\biomega}{\boldsymbol{\omega}}{d_{pc}} = 0$ were expected to be judged as colliding, the conditions with $\newcommand{\bialpha}{\boldsymbol{\alpha}}\newcommand{\bibeta}{\boldsymbol{\beta}}\newcommand{\bigamma}{\boldsymbol{\gamma}}\newcommand{\bidelta}{\boldsymbol{\delta}}\newcommand{\bivarepsilon}{\boldsymbol{\varepsilon}}\newcommand{\bizeta}{\boldsymbol{\zeta}}\newcommand{\bieta}{\boldsymbol{\eta}}\newcommand{\bitheta}{\boldsymbol{\theta}}\newcommand{\biiota}{\boldsymbol{\iota}}\newcommand{\bikappa}{\boldsymbol{\kappa}}\newcommand{\bilambda}{\boldsymbol{\lambda}}\newcommand{\bimu}{\boldsymbol{\mu}}\newcommand{\binu}{\boldsymbol{\nu}}\newcommand{\bixi}{\boldsymbol{\xi}}\newcommand{\biomicron}{\boldsymbol{\micron}}\newcommand{\bipi}{\boldsymbol{\pi}}\newcommand{\birho}{\boldsymbol{\rho}}\newcommand{\bisigma}{\boldsymbol{\sigma}}\newcommand{\bitau}{\boldsymbol{\tau}}\newcommand{\biupsilon}{\boldsymbol{\upsilon}}\newcommand{\biphi}{\boldsymbol{\phi}}\newcommand{\bichi}{\boldsymbol{\chi}}\newcommand{\bipsi}{\boldsymbol{\psi}}\newcommand{\biomega}{\boldsymbol{\omega}}{d_{pc}} = - 2$ m or $\newcommand{\bialpha}{\boldsymbol{\alpha}}\newcommand{\bibeta}{\boldsymbol{\beta}}\newcommand{\bigamma}{\boldsymbol{\gamma}}\newcommand{\bidelta}{\boldsymbol{\delta}}\newcommand{\bivarepsilon}{\boldsymbol{\varepsilon}}\newcommand{\bizeta}{\boldsymbol{\zeta}}\newcommand{\bieta}{\boldsymbol{\eta}}\newcommand{\bitheta}{\boldsymbol{\theta}}\newcommand{\biiota}{\boldsymbol{\iota}}\newcommand{\bikappa}{\boldsymbol{\kappa}}\newcommand{\bilambda}{\boldsymbol{\lambda}}\newcommand{\bimu}{\boldsymbol{\mu}}\newcommand{\binu}{\boldsymbol{\nu}}\newcommand{\bixi}{\boldsymbol{\xi}}\newcommand{\biomicron}{\boldsymbol{\micron}}\newcommand{\bipi}{\boldsymbol{\pi}}\newcommand{\birho}{\boldsymbol{\rho}}\newcommand{\bisigma}{\boldsymbol{\sigma}}\newcommand{\bitau}{\boldsymbol{\tau}}\newcommand{\biupsilon}{\boldsymbol{\upsilon}}\newcommand{\biphi}{\boldsymbol{\phi}}\newcommand{\bichi}{\boldsymbol{\chi}}\newcommand{\bipsi}{\boldsymbol{\psi}}\newcommand{\biomega}{\boldsymbol{\omega}} + 2$ m should be near-collision (thus also considered to be colliding), and the other conditions ($\newcommand{\bialpha}{\boldsymbol{\alpha}}\newcommand{\bibeta}{\boldsymbol{\beta}}\newcommand{\bigamma}{\boldsymbol{\gamma}}\newcommand{\bidelta}{\boldsymbol{\delta}}\newcommand{\bivarepsilon}{\boldsymbol{\varepsilon}}\newcommand{\bizeta}{\boldsymbol{\zeta}}\newcommand{\bieta}{\boldsymbol{\eta}}\newcommand{\bitheta}{\boldsymbol{\theta}}\newcommand{\biiota}{\boldsymbol{\iota}}\newcommand{\bikappa}{\boldsymbol{\kappa}}\newcommand{\bilambda}{\boldsymbol{\lambda}}\newcommand{\bimu}{\boldsymbol{\mu}}\newcommand{\binu}{\boldsymbol{\nu}}\newcommand{\bixi}{\boldsymbol{\xi}}\newcommand{\biomicron}{\boldsymbol{\micron}}\newcommand{\bipi}{\boldsymbol{\pi}}\newcommand{\birho}{\boldsymbol{\rho}}\newcommand{\bisigma}{\boldsymbol{\sigma}}\newcommand{\bitau}{\boldsymbol{\tau}}\newcommand{\biupsilon}{\boldsymbol{\upsilon}}\newcommand{\biphi}{\boldsymbol{\phi}}\newcommand{\bichi}{\boldsymbol{\chi}}\newcommand{\bipsi}{\boldsymbol{\psi}}\newcommand{\biomega}{\boldsymbol{\omega}}{d_{pc}} = 12$ m or $\newcommand{\bialpha}{\boldsymbol{\alpha}}\newcommand{\bibeta}{\boldsymbol{\beta}}\newcommand{\bigamma}{\boldsymbol{\gamma}}\newcommand{\bidelta}{\boldsymbol{\delta}}\newcommand{\bivarepsilon}{\boldsymbol{\varepsilon}}\newcommand{\bizeta}{\boldsymbol{\zeta}}\newcommand{\bieta}{\boldsymbol{\eta}}\newcommand{\bitheta}{\boldsymbol{\theta}}\newcommand{\biiota}{\boldsymbol{\iota}}\newcommand{\bikappa}{\boldsymbol{\kappa}}\newcommand{\bilambda}{\boldsymbol{\lambda}}\newcommand{\bimu}{\boldsymbol{\mu}}\newcommand{\binu}{\boldsymbol{\nu}}\newcommand{\bixi}{\boldsymbol{\xi}}\newcommand{\biomicron}{\boldsymbol{\micron}}\newcommand{\bipi}{\boldsymbol{\pi}}\newcommand{\birho}{\boldsymbol{\rho}}\newcommand{\bisigma}{\boldsymbol{\sigma}}\newcommand{\bitau}{\boldsymbol{\tau}}\newcommand{\biupsilon}{\boldsymbol{\upsilon}}\newcommand{\biphi}{\boldsymbol{\phi}}\newcommand{\bichi}{\boldsymbol{\chi}}\newcommand{\bipsi}{\boldsymbol{\psi}}\newcommand{\biomega}{\boldsymbol{\omega}}\infty$) were designed to be noncolliding. With NV, the subjects were able to distinguish collision (center-to-center collision and near-collision) from noncollision (Fig. 7c). Consistent with the pattern observed in our preliminary study (Qiu C, et al. *IOVS*. 2017;58:ARVO E-Abstract 3287) where similar parameters were used to script the pedestrians, a higher collision risk was perceived when the initial bearings were 10° than 30° (more noncolliding pedestrians were judged as colliding at 10° initial bearing). In addition, given $\newcommand{\bialpha}{\boldsymbol{\alpha}}\newcommand{\bibeta}{\boldsymbol{\beta}}\newcommand{\bigamma}{\boldsymbol{\gamma}}\newcommand{\bidelta}{\boldsymbol{\delta}}\newcommand{\bivarepsilon}{\boldsymbol{\varepsilon}}\newcommand{\bizeta}{\boldsymbol{\zeta}}\newcommand{\bieta}{\boldsymbol{\eta}}\newcommand{\bitheta}{\boldsymbol{\theta}}\newcommand{\biiota}{\boldsymbol{\iota}}\newcommand{\bikappa}{\boldsymbol{\kappa}}\newcommand{\bilambda}{\boldsymbol{\lambda}}\newcommand{\bimu}{\boldsymbol{\mu}}\newcommand{\binu}{\boldsymbol{\nu}}\newcommand{\bixi}{\boldsymbol{\xi}}\newcommand{\biomicron}{\boldsymbol{\micron}}\newcommand{\bipi}{\boldsymbol{\pi}}\newcommand{\birho}{\boldsymbol{\rho}}\newcommand{\bisigma}{\boldsymbol{\sigma}}\newcommand{\bitau}{\boldsymbol{\tau}}\newcommand{\biupsilon}{\boldsymbol{\upsilon}}\newcommand{\biphi}{\boldsymbol{\phi}}\newcommand{\bichi}{\boldsymbol{\chi}}\newcommand{\bipsi}{\boldsymbol{\psi}}\newcommand{\biomega}{\boldsymbol{\omega}}{\beta _{t = 0}} = 30^{\circ}$, the pedestrians designed to pass in front with $\newcommand{\bialpha}{\boldsymbol{\alpha}}\newcommand{\bibeta}{\boldsymbol{\beta}}\newcommand{\bigamma}{\boldsymbol{\gamma}}\newcommand{\bidelta}{\boldsymbol{\delta}}\newcommand{\bivarepsilon}{\boldsymbol{\varepsilon}}\newcommand{\bizeta}{\boldsymbol{\zeta}}\newcommand{\bieta}{\boldsymbol{\eta}}\newcommand{\bitheta}{\boldsymbol{\theta}}\newcommand{\biiota}{\boldsymbol{\iota}}\newcommand{\bikappa}{\boldsymbol{\kappa}}\newcommand{\bilambda}{\boldsymbol{\lambda}}\newcommand{\bimu}{\boldsymbol{\mu}}\newcommand{\binu}{\boldsymbol{\nu}}\newcommand{\bixi}{\boldsymbol{\xi}}\newcommand{\biomicron}{\boldsymbol{\micron}}\newcommand{\bipi}{\boldsymbol{\pi}}\newcommand{\birho}{\boldsymbol{\rho}}\newcommand{\bisigma}{\boldsymbol{\sigma}}\newcommand{\bitau}{\boldsymbol{\tau}}\newcommand{\biupsilon}{\boldsymbol{\upsilon}}\newcommand{\biphi}{\boldsymbol{\phi}}\newcommand{\bichi}{\boldsymbol{\chi}}\newcommand{\bipsi}{\boldsymbol{\psi}}\newcommand{\biomega}{\boldsymbol{\omega}}{d_{pc}} = + 2$ m were more likely to be perceived as colliding than those passing behind with $\newcommand{\bialpha}{\boldsymbol{\alpha}}\newcommand{\bibeta}{\boldsymbol{\beta}}\newcommand{\bigamma}{\boldsymbol{\gamma}}\newcommand{\bidelta}{\boldsymbol{\delta}}\newcommand{\bivarepsilon}{\boldsymbol{\varepsilon}}\newcommand{\bizeta}{\boldsymbol{\zeta}}\newcommand{\bieta}{\boldsymbol{\eta}}\newcommand{\bitheta}{\boldsymbol{\theta}}\newcommand{\biiota}{\boldsymbol{\iota}}\newcommand{\bikappa}{\boldsymbol{\kappa}}\newcommand{\bilambda}{\boldsymbol{\lambda}}\newcommand{\bimu}{\boldsymbol{\mu}}\newcommand{\binu}{\boldsymbol{\nu}}\newcommand{\bixi}{\boldsymbol{\xi}}\newcommand{\biomicron}{\boldsymbol{\micron}}\newcommand{\bipi}{\boldsymbol{\pi}}\newcommand{\birho}{\boldsymbol{\rho}}\newcommand{\bisigma}{\boldsymbol{\sigma}}\newcommand{\bitau}{\boldsymbol{\tau}}\newcommand{\biupsilon}{\boldsymbol{\upsilon}}\newcommand{\biphi}{\boldsymbol{\phi}}\newcommand{\bichi}{\boldsymbol{\chi}}\newcommand{\bipsi}{\boldsymbol{\psi}}\newcommand{\biomega}{\boldsymbol{\omega}}{d_{pc}} = - 2$ m (as also reported in Qiu et al.^29^). Despite the perceptual differences in collision judgment across the three viewing conditions, the subjects made comparable collision decisions for all the detected pedestrians ($\newcommand{\bialpha}{\boldsymbol{\alpha}}\newcommand{\bibeta}{\boldsymbol{\beta}}\newcommand{\bigamma}{\boldsymbol{\gamma}}\newcommand{\bidelta}{\boldsymbol{\delta}}\newcommand{\bivarepsilon}{\boldsymbol{\varepsilon}}\newcommand{\bizeta}{\boldsymbol{\zeta}}\newcommand{\bieta}{\boldsymbol{\eta}}\newcommand{\bitheta}{\boldsymbol{\theta}}\newcommand{\biiota}{\boldsymbol{\iota}}\newcommand{\bikappa}{\boldsymbol{\kappa}}\newcommand{\bilambda}{\boldsymbol{\lambda}}\newcommand{\bimu}{\boldsymbol{\mu}}\newcommand{\binu}{\boldsymbol{\nu}}\newcommand{\bixi}{\boldsymbol{\xi}}\newcommand{\biomicron}{\boldsymbol{\micron}}\newcommand{\bipi}{\boldsymbol{\pi}}\newcommand{\birho}{\boldsymbol{\rho}}\newcommand{\bisigma}{\boldsymbol{\sigma}}\newcommand{\bitau}{\boldsymbol{\tau}}\newcommand{\biupsilon}{\boldsymbol{\upsilon}}\newcommand{\biphi}{\boldsymbol{\phi}}\newcommand{\bichi}{\boldsymbol{\chi}}\newcommand{\bipsi}{\boldsymbol{\psi}}\newcommand{\biomega}{\boldsymbol{\omega}}F\left( {2,136} \right) = 0.2,\ P = 0.8$). No significant interactions were found among any factors. The decision RT (Fig. 7d) with $\newcommand{\bialpha}{\boldsymbol{\alpha}}\newcommand{\bibeta}{\boldsymbol{\beta}}\newcommand{\bigamma}{\boldsymbol{\gamma}}\newcommand{\bidelta}{\boldsymbol{\delta}}\newcommand{\bivarepsilon}{\boldsymbol{\varepsilon}}\newcommand{\bizeta}{\boldsymbol{\zeta}}\newcommand{\bieta}{\boldsymbol{\eta}}\newcommand{\bitheta}{\boldsymbol{\theta}}\newcommand{\biiota}{\boldsymbol{\iota}}\newcommand{\bikappa}{\boldsymbol{\kappa}}\newcommand{\bilambda}{\boldsymbol{\lambda}}\newcommand{\bimu}{\boldsymbol{\mu}}\newcommand{\binu}{\boldsymbol{\nu}}\newcommand{\bixi}{\boldsymbol{\xi}}\newcommand{\biomicron}{\boldsymbol{\micron}}\newcommand{\bipi}{\boldsymbol{\pi}}\newcommand{\birho}{\boldsymbol{\rho}}\newcommand{\bisigma}{\boldsymbol{\sigma}}\newcommand{\bitau}{\boldsymbol{\tau}}\newcommand{\biupsilon}{\boldsymbol{\upsilon}}\newcommand{\biphi}{\boldsymbol{\phi}}\newcommand{\bichi}{\boldsymbol{\chi}}\newcommand{\bipsi}{\boldsymbol{\psi}}\newcommand{\biomega}{\boldsymbol{\omega}}{\beta _{t = 0}} = 30^{\circ}$ was reduced in the simulated PFL condition (the interaction between the viewing condition and the initial pedestrian bearing was significant, $\newcommand{\bialpha}{\boldsymbol{\alpha}}\newcommand{\bibeta}{\boldsymbol{\beta}}\newcommand{\bigamma}{\boldsymbol{\gamma}}\newcommand{\bidelta}{\boldsymbol{\delta}}\newcommand{\bivarepsilon}{\boldsymbol{\varepsilon}}\newcommand{\bizeta}{\boldsymbol{\zeta}}\newcommand{\bieta}{\boldsymbol{\eta}}\newcommand{\bitheta}{\boldsymbol{\theta}}\newcommand{\biiota}{\boldsymbol{\iota}}\newcommand{\bikappa}{\boldsymbol{\kappa}}\newcommand{\bilambda}{\boldsymbol{\lambda}}\newcommand{\bimu}{\boldsymbol{\mu}}\newcommand{\binu}{\boldsymbol{\nu}}\newcommand{\bixi}{\boldsymbol{\xi}}\newcommand{\biomicron}{\boldsymbol{\micron}}\newcommand{\bipi}{\boldsymbol{\pi}}\newcommand{\birho}{\boldsymbol{\rho}}\newcommand{\bisigma}{\boldsymbol{\sigma}}\newcommand{\bitau}{\boldsymbol{\tau}}\newcommand{\biupsilon}{\boldsymbol{\upsilon}}\newcommand{\biphi}{\boldsymbol{\phi}}\newcommand{\bichi}{\boldsymbol{\chi}}\newcommand{\bipsi}{\boldsymbol{\psi}}\newcommand{\biomega}{\boldsymbol{\omega}}F\left( {2,136} \right) = 11.8,\ P \lt 0.0001$). Similar detection and perceived collision responses were found for the two sidewalk-like encounters (where the initial bearing was 5° or 2.5°).

Data from the eight PFL patients (Figs. 7e–h) showed response patterns similar to those seen in the simulated PFL of the normally sighted subjects, despite variations in field size and visual acuity. A two-way within-patients ANOVA (10° and 30° initial bearings × 4 path crossing distances) on the detection rate showed a significant main effect of the initial pedestrian bearing (*F*(1, 56) = 49.9, *P* < 0.0001), the path crossing distance (*F*(3, 56) = 9.8, *P* < 0.0001), and significant interaction between the two (*F*(3, 56) = 8.8, *P* < 0.0001). The detection RTs were sensitive to the initial bearing (*F*(1, 49) = 22.36, *P* < 0.0001) but not the path crossing distance (*F*(3, 49) = 2.1, *P* = 0.11) as shown in Figure 7f. Figure 7g shows that the decisions of collision by the patients are also similarly sensitive to the virtual pedestrians' trajectories (the effect of path crossing distance, *F*(3, 48) = 9.7, *P* < 0.0001). The patients' results further validated the simulator task for studying the potential impact of PFL on mobility.

### Correlations Between NV Collision Judgment and Possible Visual Cues

**Figure 8:**
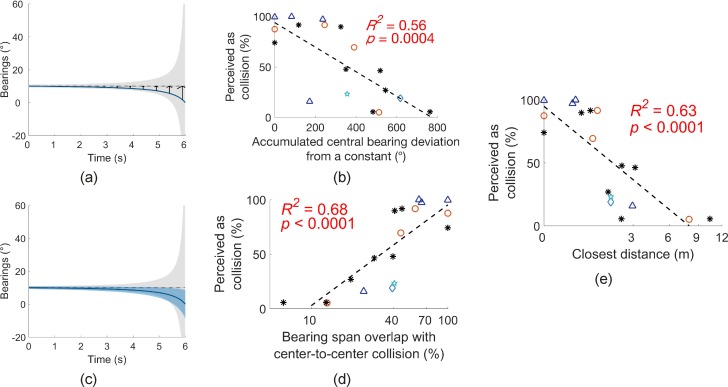
Visual cues for possible collision with an approaching pedestrian and their relationships with the collision judgment by the subjects. (a) Accumulated bearing deviations of pedestrian's central bearing from a constant bearing. The straight dashed gray line shows the constant central bearing representing the bearing of a center-to-center colliding pedestrian. The gray area indicates the bearing span of that colliding pedestrian increasing as a function of time. The dark blue curve shows the central bearing of a pedestrian passing-in-front, which starts at the same initial bearing. The black vertical arrows are samples of the accumulated bearing deviations. (b) The accumulated bearing deviations show a negative correlation with the subjects' perceived collision. (c) The bearing span overlap of the dark blue pedestrian (from (a)) with the center-to-center colliding pedestrian. The percent of overlap was calculated as the blue-gray overlapping area divided by the gray area. (d) The subjects' collision judgment shows a positive correlation with the bearing span overlap. (e) The collision judgment is negatively correlated with the closest distance between the subject and pedestrian. Data in the scatterplots are from the NV condition (same colors and icons as in Fig. 7). Additional data (black asterisks) are from (Qiu C, et al. IOVS. 2017;58:ARVO E-Abstract 3287).

A number of visual cues might be accessible while a potentially colliding pedestrian is approaching. We evaluated whether the subjects were sensitive to those cues in our virtual scenarios. First, an accumulated bearing deviation from a constant bearing was calculated using the central bearing deviation from the initial bearing for every 0.1 second from the pedestrians' initial appearance to disappearance (Fig. 8a). We found that the subject's perceived collision was significantly (and highly) correlated with the accumulated deviations of the pedestrian's central bearing from a constant. This measure accounted for about 56% of the variance in the perceived collision (Fig. 8b). Another measure of visual cue for collision is the similarity between a bearing span (apparent angular width) of an approaching pedestrian and the span of a center-to-center colliding pedestrian starting at the same initial bearing. We defined the percent of bearing span overlap: the overlapping bearing span area divided by the bearing span area of the center-to-center collision for the walk duration (Fig. 8c). The perceived collision by the subjects did show a significant positive and high correlation with the percent of bearing span overlap with the center-to-center collision, and about 68% of the variance in the perceived collision was accounted by this variable (Fig. 8d).

To evaluate whether the subjects' collision judgments were indeed reasonable, we further calculated the closest distance between the subject and the pedestrian (see Supplementary Appendix “Closest distance between the participant and pedestrian”) for each designed condition, and found that the subjects' perceived collision judgments were significantly and negatively correlated with the closest distance (Fig. 8e): given a small closest distance, the subjects were more likely to respond to the trial as collision, and the percent of perceived collision was reduced for farther closest distances. This reflected the expected natural decisions that people would make in real life. Various other factors were also explored, and the results, which were not as strongly correlated, are described in Supplementary Appendix “Visual cues and pedestrian collision judgment in normal visions.”

## Discussion

As highlighted in a Cochrane review,^37^ lack of objective outcome measures is a major failing of many vision rehabilitation clinical trials, especially for field defects. Our implementation of a novel and objective method of measuring the outcome of the field expansion devices using a VR walking simulator rather than relying on subjective questionnaires promises to be important for future clinical trials.

To determine that our VR walking scenarios are a valid testing environment, we first evaluated the impact of various conditions on the collision judgments of normally sighted subjects. Through a strong correlation between the subjects' perceived collision judgments and the calculated closest distance between the subject and the pedestrian that directly predicted whether the two would collide with each other (Fig. 8e), we verified that the virtual scenarios were indeed suitable for studying the perception of pedestrian collision. The subjects' perceived collisions among these NV conditions also showed strong correlations with the bearing deviations and the bearing span overlap (Figs. 8a–d), which are consistent with previous studies on visual cues used in collision detection^13,38–48^ (also see Supplementary Appendix “Visual cues and pedestrian collision judgment in normal vision” for more details).

We further investigated the utility of using our virtual scenarios with simulated real-life relevant tasks to measure the impact of PFL and the effect of field of view expanding prisms on performance. As expected, the eight PFL patients showed reduced pedestrian detection rates when the pedestrians initially appeared at 30° eccentricity (Fig. 6a). Their detections and decisions were also sensitive to the designed pedestrians' trajectories, as we observed in the normally sighted subjects with simulated PFL (Fig. 7), which further demonstrates the system's validity in evaluating patients' behaviors.

For the normally sighted subjects, we simulated PFL using head-mounted goggles and placed the prisms over the apertures. When pedestrians appeared outside the simulated residual central field ($\newcommand{\bialpha}{\boldsymbol{\alpha}}\newcommand{\bibeta}{\boldsymbol{\beta}}\newcommand{\bigamma}{\boldsymbol{\gamma}}\newcommand{\bidelta}{\boldsymbol{\delta}}\newcommand{\bivarepsilon}{\boldsymbol{\varepsilon}}\newcommand{\bizeta}{\boldsymbol{\zeta}}\newcommand{\bieta}{\boldsymbol{\eta}}\newcommand{\bitheta}{\boldsymbol{\theta}}\newcommand{\biiota}{\boldsymbol{\iota}}\newcommand{\bikappa}{\boldsymbol{\kappa}}\newcommand{\bilambda}{\boldsymbol{\lambda}}\newcommand{\bimu}{\boldsymbol{\mu}}\newcommand{\binu}{\boldsymbol{\nu}}\newcommand{\bixi}{\boldsymbol{\xi}}\newcommand{\biomicron}{\boldsymbol{\micron}}\newcommand{\bipi}{\boldsymbol{\pi}}\newcommand{\birho}{\boldsymbol{\rho}}\newcommand{\bisigma}{\boldsymbol{\sigma}}\newcommand{\bitau}{\boldsymbol{\tau}}\newcommand{\biupsilon}{\boldsymbol{\upsilon}}\newcommand{\biphi}{\boldsymbol{\phi}}\newcommand{\bichi}{\boldsymbol{\chi}}\newcommand{\bipsi}{\boldsymbol{\psi}}\newcommand{\biomega}{\boldsymbol{\omega}}{\beta _{t = 0}} = 30^{\circ}$), lower pedestrian detection rates with longer detection RTs were found, as we observed in the patients with PFL (Fig. 6a). As compared with the PFL patients, we also observed slightly better performance of the normally sighted subjects with simulated PFL in terms of the RTs (Fig. 6c), which may be due to their better visual acuity and younger age. It may also be affected by the visibility of the PFL. The edges of the simulated PFL apertures are visible and increase the subjects' awareness, while for the patients there is no such apparent visual clue. The detection rates in the normally sighted subjects with simulated PFL ranged from 47% to 97% (Fig. 7a) and in the patients from 27% to 90% (Fig. 7e), depending on the path crossing distance. These high detection rates even with very limited fields may be accounted for by two factors: first, for the cases with $\newcommand{\bialpha}{\boldsymbol{\alpha}}\newcommand{\bibeta}{\boldsymbol{\beta}}\newcommand{\bigamma}{\boldsymbol{\gamma}}\newcommand{\bidelta}{\boldsymbol{\delta}}\newcommand{\bivarepsilon}{\boldsymbol{\varepsilon}}\newcommand{\bizeta}{\boldsymbol{\zeta}}\newcommand{\bieta}{\boldsymbol{\eta}}\newcommand{\bitheta}{\boldsymbol{\theta}}\newcommand{\biiota}{\boldsymbol{\iota}}\newcommand{\bikappa}{\boldsymbol{\kappa}}\newcommand{\bilambda}{\boldsymbol{\lambda}}\newcommand{\bimu}{\boldsymbol{\mu}}\newcommand{\binu}{\boldsymbol{\nu}}\newcommand{\bixi}{\boldsymbol{\xi}}\newcommand{\biomicron}{\boldsymbol{\micron}}\newcommand{\bipi}{\boldsymbol{\pi}}\newcommand{\birho}{\boldsymbol{\rho}}\newcommand{\bisigma}{\boldsymbol{\sigma}}\newcommand{\bitau}{\boldsymbol{\tau}}\newcommand{\biupsilon}{\boldsymbol{\upsilon}}\newcommand{\biphi}{\boldsymbol{\phi}}\newcommand{\bichi}{\boldsymbol{\chi}}\newcommand{\bipsi}{\boldsymbol{\psi}}\newcommand{\biomega}{\boldsymbol{\omega}}{d_{pc}} = + 2$ m or $\newcommand{\bialpha}{\boldsymbol{\alpha}}\newcommand{\bibeta}{\boldsymbol{\beta}}\newcommand{\bigamma}{\boldsymbol{\gamma}}\newcommand{\bidelta}{\boldsymbol{\delta}}\newcommand{\bivarepsilon}{\boldsymbol{\varepsilon}}\newcommand{\bizeta}{\boldsymbol{\zeta}}\newcommand{\bieta}{\boldsymbol{\eta}}\newcommand{\bitheta}{\boldsymbol{\theta}}\newcommand{\biiota}{\boldsymbol{\iota}}\newcommand{\bikappa}{\boldsymbol{\kappa}}\newcommand{\bilambda}{\boldsymbol{\lambda}}\newcommand{\bimu}{\boldsymbol{\mu}}\newcommand{\binu}{\boldsymbol{\nu}}\newcommand{\bixi}{\boldsymbol{\xi}}\newcommand{\biomicron}{\boldsymbol{\micron}}\newcommand{\bipi}{\boldsymbol{\pi}}\newcommand{\birho}{\boldsymbol{\rho}}\newcommand{\bisigma}{\boldsymbol{\sigma}}\newcommand{\bitau}{\boldsymbol{\tau}}\newcommand{\biupsilon}{\boldsymbol{\upsilon}}\newcommand{\biphi}{\boldsymbol{\phi}}\newcommand{\bichi}{\boldsymbol{\chi}}\newcommand{\bipsi}{\boldsymbol{\psi}}\newcommand{\biomega}{\boldsymbol{\omega}} + 12$ m, the pedestrians passed in front of the participant and thus eventually entered the residual central field to be detected. The detection RTs for these cases were therefore expected to be longer, which is exactly what our results of detection RT indicate (Figs. 7b, 7f). Second, some participants may adopt a scanning strategy, given the regularity of the task and additionally for normally sighted subjects the visibility of the field obstruction. The simulated PFL also led to reduced decision RTs (Fig. 7d) compared with the NV condition; and the patients showed the shortest decision RTs (Fig. 6c). It is likely that the participants spent a longer time on detection; therefore, by the time the pedestrian was detected, the collision/noncollision event was so imminent that it required little time to judge and respond.

Importantly, the peripheral prisms did not impede pedestrian detection or collision judgment for events within the residual central field. In the prism-expanded field, the prisms increased pedestrian detection rate, decreased the detection RT, and did not negatively affect the accuracy of the collision judgment. We expect that the prisms will be easier to adapt to with wider/taller residual central islands that enable the positioning of the prisms farther in the periphery, and that may facilitate better adaptation during later phases of disease progression, where the residual central island is reduced in size.

With that verification of the evaluating system and support of the PFL patients' data, we are confident that we can pursue future studies on the effect of the prisms and other possible treatments using this paradigm. We also plan to evaluate the utility of the naturally occurring nasal residual islands in patients with RP for detecting approaching pedestrians on a collision course. It was recently reported^1^ that these natural islands are often centered at the eccentricity of about 45°, where the risk density for collision with another pedestrian in an open space environment peaks. To our knowledge, there are no studies addressing the utility of these residual islands for any purpose, and certainly not for the avoidance of collisions.

Although the study verifies the virtual environment for PFL testing and demonstrates benefits of the peripheral prisms for simulated PFL in detecting colliding pedestrians, there are limitations to be considered. First, the participants were sitting, not actually walking (with the associated head bobbing), and their attention is directed to the ball tracking (though this may resemble the necessary attentional load for wayfinding and front obstacle avoidance). Second, while the use of frequent pedestrians provides effective repeated trial evaluation, it might have primed the participants and increased their anticipation of the events, which possibly elicited frequent scanning. Last, the current scenarios lacked the complexity of multiple collision risks that may be present simultaneously in crowded spaces. Although the participants were required to detect pedestrians against a natural background with trees and buildings, detecting pedestrians in real-life scenes may be even more challenging. Note also that the addition of other pedestrians or obstacles may go unnoticed as these are likely to appear and stay outside of the residual field (natural and expanded) for most of the trial. This is why it is important to aim the expansion at the highest risk eccentricity.

In addition, the prisms with simulated PFL do not exactly simulate the prisms on glasses lens. The longer vertex distance required to avoid diffraction reduces prism effectivity from 30° (≈57Δ) to 26° (≈49Δ). Also, the subjects were instructed to try not to scan with their eyes inside the goggles. Such scanning has no central field of view benefit in the goggles simulation, which is not true for patients with real PFL. Note that due to the prism TIR, eye scanning would not achieve further prism field of view expansion either in the simulations or the patients' glasses. Therefore, devices that are less affected by the TIR are likely to be more beneficial.^25^

## Summary

We verified that the walking scenarios rendered in a virtual environment were practical for evaluating the effect of PFL and the impact of field of view expanding prisms in pedestrian detection and collision judgment. The current design of peripheral prisms for PFL has demonstrated substantial benefits in pedestrian detection (despite small apertures and the inevitable binocular visual confusion associated with prismatic field expansion) and shown minimal interference with the residual central field. Future work will include further evaluation of patients' residual peripheral fields and the impact of prisms mounted on glasses lens.

## Supplementary Material

Supplement 1

## References

[1] Peli E, Apfelbaum H, Berson EL, Goldstein RB (2016). The risk of pedestrian collisions with peripheral visual field loss. *J Vis*.

[2] Apfelbaum H, Peli E (2015). Tunnel vision prismatic field expansion: challenges and requirements. *Transl Vis Sci Technol*.

[3] U.S. Social Security Administration (2017). Disability evaluation under social security. https://www.ssa.gov/disability/professionals/bluebook/2.00-SpecialSensesandSpeech-Adult.htm#2_02.

[4] Grover S, Fishman GA, Anderson RJ, Alexander KR, Derlacki DJ (1997). Rate of visual field loss in retinitis pigmentosa. *Ophthalmology*.

[5] Massof RW, Dagnelie G, Benzschawel T, Palmer RW, Stein DF (1990). First order dynamics of visual field loss in retinitis pigmentosa. *Clin Vis Sci*.

[6] Turano KA, Geruschat DR, Stahl JW, Massof RW (1999). Perceived visual ability for independent mobility in persons with retinitis pigmentosa. *Invest Ophthalmol Vis Sci*.

[7] Geruschat DR, Turano KA, Stahl JW (1998). Traditional measures of mobility performance and retinitis pigmentosa. *Optom Vis Sci*.

[8] Lovie-Kitchin J, Mainstone J, Robinson J, Brown B (1990). What areas of the visual field are important for mobility in low vision patients?. *Clin Vis Sci*.

[9] Lovie-Kitchin JE, Soong GP, Hassan SE, Woods RL (2010). Visual field size criteria for mobility rehabilitation referral. *Optom Vis Sci*.

[10] Haymes S, Guest D, Heyes A, Johnston A (1996). Mobility of people with retinitis pigmentosa as a function of vision and psychological variables. *Optom Vis Sci*.

[11] Kuyk T, Elliot JL, Fuhr PW (1998). Visual correlates of mobility in real world settings in older adults with low vision. *Optom Vis Sci*.

[12] Iorizzo DB, Riley ME, Hayhoe M, Huxlin KR (2011). Differential impact of partial cortical blindness on gaze strategies when sitting and walking: an immersive virtual reality study. *Vision Res*.

[13] Regan D, Gray R (2001). Hitting what one wants to hit and missing what one wants to miss. *Vision Res*.

[14] Hollands MA, Patla AE, Vickers JN (2002). Look where you're going!: gaze behaviour associated with maintaining and changing the direction of locomotion. *Exp Brain Res*.

[15] Regan D, Beverley KI (1982). How do we avoid confounding the direction we are looking and the direction we are moving?. *Science*.

[16] Geruschat DR, Turano KA (2002). Connecting research on retinitis pigmentosa to the practice of orientation and mobility. *J Vis Impair Blind*.

[17] Luo G, Vargas-Martin F, Peli E (2008). The role of peripheral vision in saccade planning: Learning from people with tunnel vision. *J Vis*.

[18] Vargas-Martin F, Peli E (2006). Eye movements of patients with tunnel vision while walking. *Invest Ophthalmol Vis Sci*.

[19] Peli E, Jung JH (2017). Multiplexing prisms for field expansion. *Optom Vis Sci*.

[20] Peli E (2000). Field expansion for homonymous hemianopia by optically-induced peripheral exotropia. *Optom Vis Sci*.

[21] Peli E (2001). Vision multiplexing: an engineering approach to vision rehabilitation device development. *Optom Vis Sci*.

[22] Apfelbaum HL, Ross NC, Bowers AB, Peli E (2013). Considering apical scotomas, confusion, and diplopia when prescribing prisms for homonymous hemianopia. *Transl Vis Sci Technol*.

[23] Bowers A, Keeney K, Peli E (2014). Randomized crossover clinical trial of real and sham peripheral prism glasses for hemianopia. *JAMA Ophthalmol*.

[24] Peli E, Bowers AR, Keeney K, Jung JH (2016). High-power prismatic devices for oblique peripheral prisms. *Optom Vis Sci*.

[25] Jung JH, Peli E (2014). Impact of high power and angle of incidence on prism corrections for visual field loss. *Opt Eng*.

[26] Houston KE, Peli E, Goldstein RB, Bowers AR (2018). Driving with hemianopia VI: peripheral prims and perceptual-motor training improve blind-side detection in a driving simulator. *Transl Vis Sci Technol*.

[27] Bowers AR, Tant M, Peli E (2012). A pilot evaluation of on-road detection performance by drivers with hemianopia using oblique peripheral prisms. *Stroke Res Treat*.

[28] Luo G, Peli E (2006). Use of an augmented-vision device for visual search by patients with tunnel vision. *Invest Ophthalmol Vis Sci*.

[29] Qiu C, Spano LP, Tuccar-Burak M, Goldstein R, Jung JH, Peli E (2016). Judging pedestrian collisions in open-space walking simulations (Abstract). *Academy 2016*.

[30] Apfelbaum HL, Pelah A, Peli E (2007). Heading assessment by “tunnel vision” patients and control subjects standing or walking in a virtual reality environment. *ACM Trans Applied Perception*.

[31] Harris MG, Carre G (2001). Is optic flow used to guide walking while wearing a displacing prism?. *Perception*.

[32] Rushton SK, Harris JM, Lloyd MR, Wann JP (1998). Guidance of locomotion on foot uses perceived target location rather than optic flow. *Curr Biol*.

[33] Warren WH, Kay BA, Zosh WD, Duchon AP, Sahuc S (2001). Optic flow is used to control human walking. *Nat Neurosci*.

[34] Woods RL, Giorgi RG, Berson EL, Peli E (2010). Extended wearing trial of Trifield lens device for “tunnel vision”. *Ophthalmic Physiol Opt*.

[35] Greenhouse SW, Geisser S (1959). On methods in the analysis of profile data. *Psychometrika*.

[36] Holm S (1979). A simple sequentially rejective multiple test procedure. *Scand StatTheory Appl*.

[37] Pollock A, Hazelton C, Henderson CA (2011). Interventions for visual field defects in patients with stroke. *Cochrane Database Syst Rev*.

[38] Gray R, Regan D (2000). Simulated self-motion alters perceived time to collision. *Curr Biol*.

[39] Regan D, Vincent A (1995). Visual processing of looming and time to contact throughout the visual field. *Vision Res*.

[40] Bootsma RJ, Craig CM (2003). Information used in detecting upcoming collision. *Perception*.

[41] Tresilian JR (1991). Empirical and theoretical issues in the perception of time to contact. *J Exp Psychol Hum Percept Perform*.

[42] Bootsma RJ, Oudejans RR (1993). Visual information about time-to-collision between two objects. *J Exp Psychol Hum Percept Perform*.

[43] Kaiser MK, Mowafy L (1993). Optical specification of time-to-passage: Observers' sensitivity to global tau. *J Exp Psychol Hum Percept Perform*.

[44] Yan J-J, Lorv B, Li H, Sun H-J (2011). Visual processing of the impending collision of a looming object: time to collision revisited. *J Vis*.

[45] DeLucia PR, Meza-Arroyo M, Baurès R, Ranjit M, Hsiang S, Gorman JC (2016). Continuous response monitoring of relative time-to-contact judgments: does effective information change during an approach event?. *Ecologic Psych*.

[46] Todd JT (1981). Visual information about moving objects. *J Exp Psychol Hum Percept Perform*.

[47] Fajen BR (2013). Guiding locomotion in complex, dynamic environments. *Front Behav Neurosci*.

[48] Pundlik S, Peli E, Luo G, Bebis G, Boyle R, Parvin B (2011). Time to collision and collision risk estimation from local scale and motion. *Advances in Visual Computing*.

[49] Fry GA (1978). Face-form frames. *J Am Optom Assoc*.

[50] Cutting JE, Vishton PM, Braren PA (1995). How we avoid collisions with stationary and moving obstacles. *Psychol Rev*.

[51] Andersen GJ, Kim RD (2001). Perceptual information and attentional constraints in visual search of collision events. *J Exp Psychol Hum Percept Perform*.

