# Supplementary material for: Measuring Pedestrian Collision Detection With Peripheral Field Loss and the Impact of Peripheral Prisms
Source: Transl Vis Sci Technol. 2018 Sep 4;7(5):1. doi: 10.1167/tvst.7.5.1 (PMC6126965; doi:10.1167/tvst.7.5.1)
Supplement: Supplement 1 [file tvst-07-03-19_s01.pdf]

Appendix

Details of prism positioning

Rigid polymethyl methacrylate (PMMA) Fresnel prisms with the currently highest commercially available power, 57Δ, are embedded into the upper and lower peripheral portions of the spectacle lenses, which are customized for each patient's residual central field size. An outward prism serration (OPS) configuration is used to maximize the effective prism power and thus the magnitude of the field of view expansion.[25] For a given prism location (upper or lower), a unilateral placement has the prism only in front of one eye to achieve *binocular multiplexing* in visual field expansion.[21] In both locations, the prisms are placed base-out, with the right lens prism expanding the field to the right and the left lens prism to the left. The base-out placement further takes advantage of the spectacle face-form frame tilt[49] to reduce the limitation of prism total internal reflection (TIR).[25] In the region of TIR, no expanded views are seen and instead spurious reflections may be visible.[25] With a 57Δ OPS prism placed in a fronto-parallel plane, the TIR starts at about 5° eccentricity towards the prism base, which is well within the patient's residual central field, as illustrated in Fig. 5b to occur even at primary gaze. Therefore, the TIR should be minimized, especially with PFL patients.

With the prism placed in the fronto-parallel plane (Fig. A1a), the angle of incidence is same as the visual eccentricity and the TIR starts at 5° visual eccentricity on the base side as the prism power increases with the visual eccentricity toward the base side. However, the face-form tilt[49] can reduce (base-out, Fig. A1b) or increase (base-in, Fig. A1c) the magnitude of the angle of incidence toward the base side even at the same visual eccentricity. When the peripheral prisms are placed on the spectacles in a base-out configuration, the face-form tilt of the frame[49]

reduces the absolute angle of incidence on the base side and consequently moves the TIR transition point farther peripherally (Fig. A1b). On the other hand, in base-in configuration, the face-form tilt increases the absolute angle of incidence on the base side, which results in moving the TIR transition point centrally (Fig. A1c).

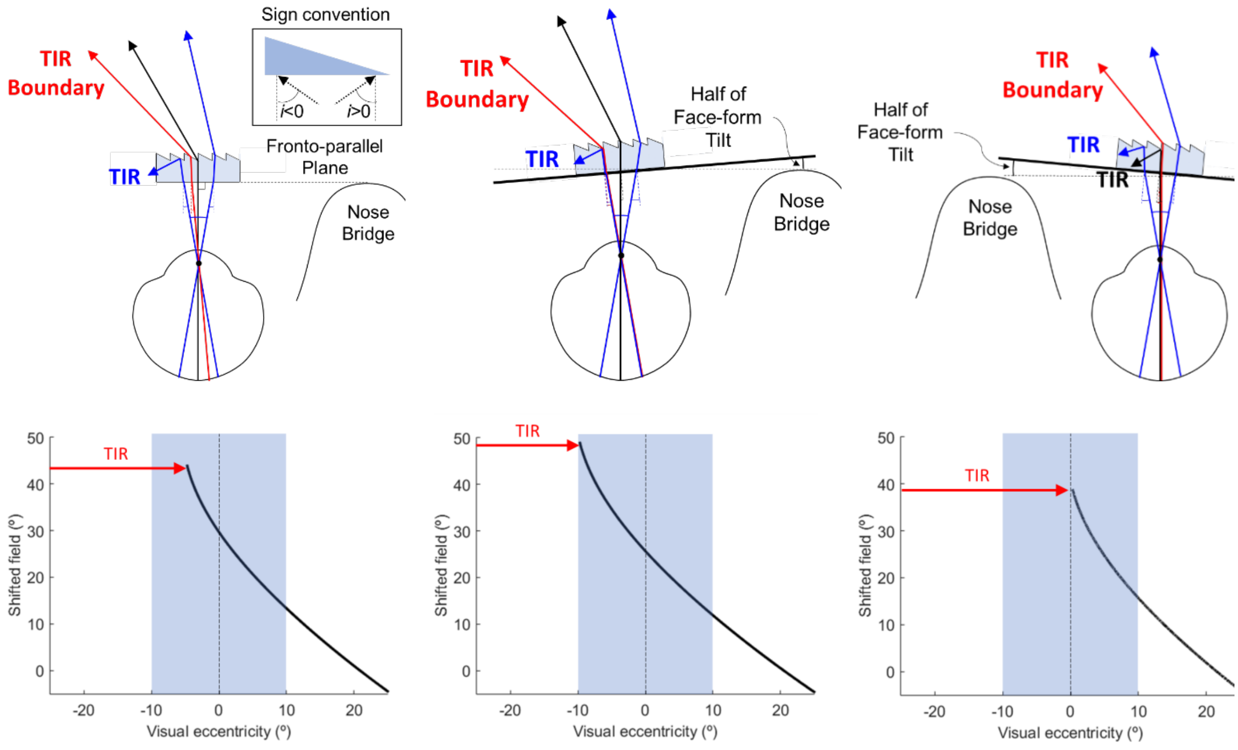

(a) (b) (c)

Figure A1. Effect of face-form tilt in various prism base configurations. (a) Top, view from above of the ray diagram when the prisms are placed in fronto-parallel plane with view at primary gaze (black arrow). The angle of incidence in red indicates the critical angle of incidence where TIR first occurs. Blue arrows indicate the residual central field in the patient with PFL. In left HH, the left side of the primary gaze (black arrow) is blind. The graph below shows the shifted field as a function of visual eccentricity with 57Δ base-out prism. Due to the TIR at about -5°, eccentricities farther than that cannot be seen in the shifted field. The residual central field is indicated in blue highlighted area and left of the vertical dashed line is the blind side of the patient with left HH. When the total face-form tilt is 10° (5° on each side), ray diagrams and the graphs for (b) base-out and (c) base-in prism are shown. Due to the decreased magnitude of angle of incidence in the base-out configuration by the face-form tilt, TIR is moved farther to larger visual eccentricity and does not impede visibility within the residual central field of the patient with PFL in primary position of gaze (also providing for wider eye scanning range in HH). However, in base-in configuration, TIR blocks vision for almost half of the residual central field and provides no eye scanning range in HH. In addition,

the base-out configuration in (b) shows much farther field expansion in the blind side than the other two configurations.

For a HH patient with the eyes at the primary position of gaze, that range of ineffective expansion is usually in the blind field area, and does not affect the functionality of the prisms. The only impact occurs when the patient scans toward the blind side, where for eye movements larger than 5º the fovea direction is pointed into TIR range (though below it). As a result, the peripheral field expansion does not increase beyond the expansion achieved with 5º scanning.[25] However, in PFL with its residual field around the fovea, the TIR area is likely to fall within the residual central field even in primary position of gaze and limit the field expansion effect of the high-power prisms, as shown in Fig 5b. In addition, image compression is higher near the TIR transition and thus visibility is lower due to the minification. Therefore, the transition to the TIR region should be pushed towards the blind field as much as possible. Since the field loss surrounds the residual central island, the prism TIR would be pushed farther out by the face-form tilt if the placement is always in a base-out configuration. With the base-in configuration, the field expansion is limited to only about half of the span of the residual central island and further limits any benefit of eye scanning.[24]

Peripheral islands created by the prisms

The longer back vertex distance used in the goggles ensured an accurate simulation of the residual central field size, but slightly reduced prism effectivity. The prism shift happens at the prism. When the prism is close to the eye (i.e., about 16 mm from the entrance pupil of the eye in prism glasses), the prism shift (prism power) and perceived shift (perceived prism power) are approximately same. However, in the goggles we used in this paper, due to large back vertex distance (100mm), the back vertex distance on prism power should be considered.

The 57Δ prism deflection angle is: $\tan^{-1}\left(\frac{57}{100}\right) = 29.7°$. The distance between the driving simulator screens and the cornea was 735 mm. In the conventional prism glasses (13 mm back vertex distance), the prism on the spectacle shows (735 – 13) × tan (29.7°) = 411.8 mm into the base side of the screen at the primary gaze, which is approximately same as the perceived shift $\tan^{-1}\left(\frac{411.8}{735+3}\right) = 29.2°$.

However, in the goggles with long back vertex distance (100 mm), the prism on the goggles shows (735 – 100) × tan (29.7°) = 366.2 mm into the base side of the screen at the primary gaze, which results in only $\tan^{-1}\left(\frac{362.2}{735+3}\right) = 26.1° \approx 49\ \Delta$ in the perceived shift.

To bring the center of the shifted views vertically toward eye level, we use an oblique prism that provides a 7.5° vertical shift. The tilted angle of the prism apex-base axis, t, should be calculated by solving the equation, $26.1° \times \sin(t) = 7.5°$, which gives $t = 16.7°$. As shown in Fig. 4a the prisms were rotated by about 17° so that the apex to base axis pointed toward the eye level. This rotation, however, resulted in a reduced horizontal effective angle deviation to $26.1° \times \cos(16.7) = 25.0°$ (≈ 46 Δ).

For high-power prisms, the prism power is not constant and varies with angle of incidence.[25] The prism placed on the goggles has a combination of effect of oblique tilt, angle of incidence, and perceived prism shift from the long back vertex distance. Since oblique prisms were used on the goggles, the angle of incidence is also affected by the oblique tilt angle. Therefore, ±8.7° visual eccentricities in the residual central field create $\pm 8.7° \times \cos(17°) = \pm 8.3°$ angles of incidence. Due to the -4.7° critical angle of incidence at 57Δ prism with 50% transmittance,[25] TIR starts at -4.9° visual eccentricity from $-4.9° \times \cos(17°) = -4.7°$ relation

in the oblique prism. Therefore, the range of visual eccentricity is from -4.9° to +8.7°, which result in angles of incidence from -4.7° to +8.3°.

Since prism power at -4.7° and 8.3° angle of incidence are 40.3° and 22.8° respectively,[25] the reduced lateral shift in oblique design is 38.5° and 21.8°, respectively. Therefore, the prism shows in the spectacles the island from 13° (=21.8 – 8.7) to 43° (=38.5 + 4.9) which results in the perceived lateral shift from 11° to 39° due to the long vertex distance in the goggles.

Closest distance between the participant and pedestrian

We calculated the closest distance between two people while they are approaching to determine whether such distance is larger than their body width so they could pass without contact. As illustrated in Fig. A2, the participant starting (at $t = 0$) from an initial position at $(m_0,\ n_0)$ and a speed of $\overrightarrow{v_s} = (s_1, s_2)$; the pedestrian starting from an initial position $(x_0,\ y_0)$ with $\overrightarrow{v_p} = (p_1, p_2)$. Since only the relative positions were of interest, for convenience we set the participant starting position at the origin $(m_0 = n_0 = 0)$ and headed toward the north $(s_1 = 0)$. At time t, the participant would be at the position of $(0,\ s_2 t)$, and the pedestrian at $(x_0 + p_1 t,\ y_0 + p_2 t)$. The distance d between them at time t is given by

$$d^2 = [(x_0 + p_1 t) - 0]^2 + [(y_0 + p_2 t) - s_2 t]^2. \qquad \text{(A1)}$$

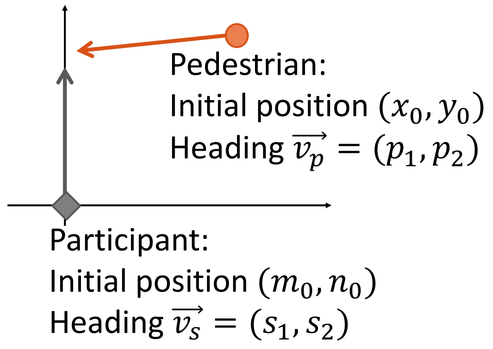

Figure A2. The distance between the participant and the pedestrian at time *t* is specified, and the closest distance between them while approaching is then calculated.

Rewriting equation A1 as a function of time t,

$$d^2 = [p_1^2 + (p_2 - s_2)^2]t^2 + 2\big(x_0p_1 + y_0(p_2 - s_2)\big)t + (x_0^2 + y_0^2). \qquad \text{(A2)}$$

When $p_1^2 + (p_2 - s_2)^2 = 0$, that is, $p_1 = 0$ and $p_2 = s_2$, the participant and the pedestrian would both walk toward north with the same speed, and the distance between them becomes a constant $d = \sqrt{x_0^2 + y_0^2}$ (since the second part of the equation $2\big(x_0p_1 + y_0(p_2 - s_2)\big) = 0$ as well); When $p_1^2 + (p_2 - s_2)^2 > 0$, d^2 is equal to a polynomial in t of degree 2. The minimum, that is, the closest distance, $d^2 = (x_0^2 + y_0^2) - \frac{x_0^2p_1^2 + y_0^2(p_2-s_2)^2 + 2x_0y_0p_1(p_2-s_2)}{p_1^2 + (p_2-s_2)^2}$, would be reached when $t = -\frac{x_0p_1 + y_0(p_2-s_2)}{p_1^2 + (p_2-s_2)^2}$. These were calculated for each pedestrian and the results correlated with participants' perceived collision judgment as shown in Fig. 8e.

Visual cues and pedestrian collision judgment in normal vision (NV)

Besides the bearing deviation, bearing span overlap, and the closest distance, we also tested the relationship between the collision judgment and other factors such as pedestrians' initial bearing and speed. We found that the subject's perceived collision showed no significant correlation with the pedestrian's initial bearing relative to the subject's heading (Fig. A3a). The pedestrian's speed had a statistically significant but only modest correlation with the perceived collision (Fig. A3b). On the other hand, the time when the closest distance was reached was strongly correlated with the subject's overall response time (detection RT + decision RT), although the latter was always shorter (Fig. A3c).

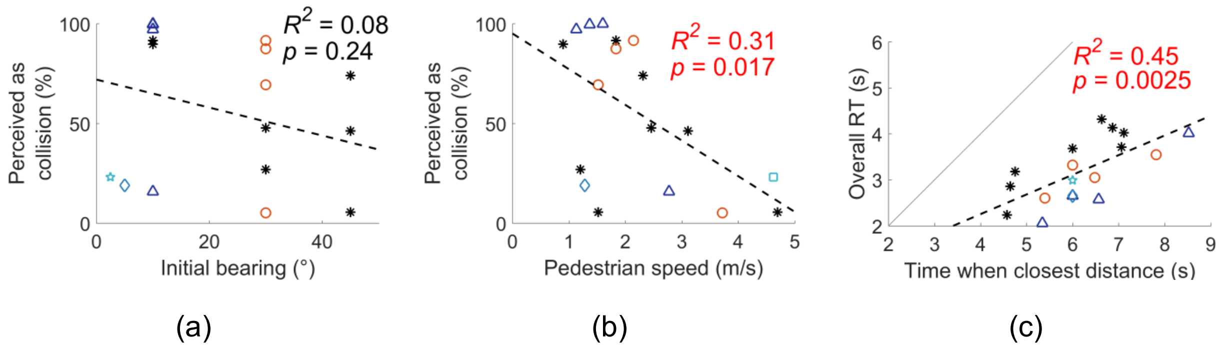

Figure A3. Scatterplots for the data from the normal vision (NV) condition (including additional data from (Qiu C, et al. IOVS 2017;58:ARVO E-Abstract 3287), shown as black asterisks. For data acquired in the current experiment, the same colors and icons are used as in Fig. 7. The percent of perceived collision as a function of (a) pedestrian's initial bearing relative to the subject's heading and (b) pedestrian's speed. (c) The overall response time (detection RT + decision RT) as a function of the time when the closest distance between the subject and pedestrian occurs.

The closest distance, however, is only directly available to the subject when the collision/near-collision is imminent, which will always lead to an untimely response. Although we did find a correlation between the overall response time and the time when the closest distance is reached, the former was considerably shorter than the latter (Fig. A3c), indicating that subjects rely on other visual cues to make effective collision judgments before reaching the closest distance.

Various visual cues have been studied in the literature. For example, the time-to-collision (TTC), which could be inferred based on the approaching object's angular subtense and its optical expansion rate (looming), is thought to be a reliable cue for collision detection.[13, 38-48] Tresilian[41] further pointed out that if the potential colliding object appeared off to the side (with a non-zero bearing), the rate of the bearing changes would also influence the collision perception (also see[42, 43, 50]). In a center-to-center collision, the bearing of the pedestrian's central point would not change; that is, it would stay constant (as shown in Fig. 1d-f). To characterize the

changes in the bearing of the center, we calculated the accumulated bearing deviation from a constant (Fig. 8a), which explained about 56% of the variance in the perceived collision response data (Fig. 8b). The larger the accumulated deviation, the less likely the subject is to judge the pedestrian as colliding.

Both constant bearing and looming were further analyzed in collision perception literature.[42, 43, 51] To see how well the conjunction of these cues would predict subjects' responses in our experiments, we used a measure of bearing span overlap with a center-to-center collision (Fig. 8c). The visual pattern of a pedestrian on a center-to-center collision course with the subject showed that the pedestrian's bearing span would expand relative to a constant central bearing (as in Fig. 1f). The more similar the visual pattern of an approaching pedestrian is to the center-to-center collision, the more likely the pedestrian would be judged as colliding. Therefore, a large percent of overlap indicated a potential collision (center-to-center and near-collision). This measure indeed explained 68% of the variance in our collision judgment responses (Fig. 8d). The collision decisions did not seem to be well correlated with other factors, such as pedestrian speed (only 31% of the variance, Fig. A3b) or pedestrians' initial bearings (8% of the variance, Fig. A3a).
